# Distinct epitope structures of defensin‐like proteins linked to proline‐rich regions give rise to differences in their allergenic activity

**DOI:** 10.1111/all.13298

**Published:** 2017-09-27

**Authors:** I. Pablos, S. Eichhorn, Y. Machado, P. Briza, A. Neunkirchner, B. Jahn‐Schmid, S. Wildner, W. T. Soh, C. Ebner, J.‐W. Park, W. F. Pickl, N. Arora, S. Vieths, F. Ferreira, G. Gadermaier

**Affiliations:** ^1^ Division of Allergy and Immunology Department of Molecular Biology University of Salzburg Salzburg Austria; ^2^ Center for Pathophysiology, Infectiology and Immunology Institute of Immunology Medical University of Vienna Vienna Austria; ^3^ Department of Pathophysiology and Allergy Research Medical University of Vienna Vienna Austria; ^4^ Christian Doppler Laboratory for Biosimilar Characterization University of Salzburg Salzburg Austria; ^5^ Allergy Clinic Reumannplatz Vienna Austria; ^6^ Department of Internal Medicine and Institute of Allergy Yonsei University College of Medicine Seoul Korea; ^7^ Allergy and Immunology Section CSIR‐Institute of Genomic and Integrative Biology Delhi India; ^8^ Division of Allergology Paul‐Ehrlich‐Institut Langen Germany; ^9^ Present address: Department of Oral Biological and Medical Science University of British Columbia Vancouver Canada

**Keywords:** allergens and epitopes, Amb a 4, Art v 1, defensin‐like allergen, weed pollen allergy

## Abstract

**Background:**

Art v 1, Amb a 4, and Par h 1 are allergenic defensin‐polyproline–linked proteins present in mugwort, ragweed, and feverfew pollen, respectively. We aimed to investigate the physicochemical and immunological features underlying the different allergenic capacities of those allergens.

**Methods:**

Recombinant defensin‐polyproline–linked proteins were expressed in *E. coli* and physicochemically characterized in detail regarding identity, secondary structure, and aggregation status. Allergenic activity was assessed by mediator releases assay, serum IgE reactivity, and IgE inhibition ELISA using sera of patients from Austria, Canada, and Korea. Endolysosomal protein degradation and T‐cell cross‐reactivity were studied in vitro.

**Results:**

Despite variations in the proline‐rich region, similar secondary structure elements were observed in the defensin‐like domains. Seventy‐four percent and 52% of the Austrian and Canadian patients reacted to all three allergens, while Korean patients were almost exclusively sensitized to Art v 1. This was reflected by IgE inhibition assays demonstrating high cross‐reactivity for Austrian, medium for Canadian, and low for Korean sera. In a subgroup of patients, IgE reactivity toward structurally altered Amb a 4 and Par h 1 was not changed suggesting involvement of linear epitopes. Immunologically relevant endolysosomal stability of the defensin‐like domain was limited to Art v 1 and no T‐cell cross‐reactivity with Art v 1_25‐36_ was observed.

**Conclusions:**

Despite structural similarity, different IgE‐binding profiles and proteolytic processing impacted the allergenic capacity of defensin‐polyproline–linked molecules. Based on the fact that Amb a 4 demonstrated distinct IgE‐binding epitopes, we suggest inclusion in molecule‐based allergy diagnosis.

## 1 INTRODUCTION

Allergy to weed pollen has been extensively documented worldwide, being the third most important cause of pollen allergies, after grasses and trees. Relevant allergenic weeds of the Asteraceae family are mugwort *(Artemisia vulgaris)*, ragweed *(Ambrosia artemisiifolia)*, Santa Maria feverfew *(Parthenium hysterophorus),* and sunflower *(Helianthus annuus)*.^1^ Mugwort and ragweed are preferentially growing in the temperate climate zone of the Northern Hemisphere and Australia, while feverfew grows in the subtropical areas of America and Asia. In Europe, exposure to mugwort pollen is more abundant in central and northern regions, ragweed in south and eastern areas, while feverfew is not endemic (www.pollenwarndienst.at). In northern America, ragweed represents the most prevalent Asteraceae pollen source, while in Korea exposure to mugwort and ragweed is observed.^2^, ^3^ Atypical distribution and intensified flowering seasons of weeds are anticipated which potentially lead to more allergic reactions as a consequence of climatic changes.^4^, ^5^, ^6^

Extract‐based allergy diagnosis of weed pollen is frequently challenging due to (i) polysensitization of patients, (ii) overlapping flowering periods, and (iii) similar allergen profiles.^7^, ^8^ Although molecule‐based approaches can be exploited for diagnosis, solely Amb a 1, Art v 1, and Art v 3 are currently available for routine allergy diagnosis of Asteraceae pollen allergy.^2^ Several studies showed that sensitization profiles to distinct allergens from the same weed are highly diverse in different geographic regions.^4^, ^7^, ^9^, ^10^ Even molecules from the same protein family like lipid transfer proteins (LTP) or pectate lyases can show different sensitization potencies, and in this context, allergenic defensin‐like proteins linked to a polyproline‐rich region are interesting molecules. Those defensin‐polyproline–linked proteins are exclusively found in pollen of the Asteraceae family, while defensin‐like proteins alone are prevalent in higher plants and were recently also described as allergens in legumes.^1^, ^11^, ^12^

Art v 1 is a major allergen with a sensitization rate of 95% among mugwort pollen‐allergic patients.^13^ Amb a 4, the homolog in ragweed, is considered a minor allergen with 20%‐40% sensitization rate among ragweed‐allergic patients.^14^ Recently, the complete sequence of Par h 1 from feverfew was identified and a sensitization rate of 60% and 40% among Austrian and Indian pollen‐allergic patients was reported.^15^ A common feature of this allergen family is O‐linked glycosylation of hydroxyproline residues.^13^, ^14^, ^16^, ^17^ Despite the fact that low levels of IgE antibodies against glycan moieties of Art v 1 were detected, they did not convey mediator release and thus demonstrated low clinical significance.^18^ If O‐linked glycans contribute to unspecific cross‐reactivity in diagnosis remains to be determined. While structural and immunological features of Art v 1 have been extensively investigated,^13^, ^16^, ^19^, ^20^ only limited information is available for Amb a 4 and Par h 1.^14^, ^15^

In the present study, we investigated structural and immunological features of allergenic defensin‐polyproline–linked molecules from mugwort, ragweed, and feverfew pollen. Therefore, recombinant allergens were produced to evaluate intrinsic physicochemical characteristics. IgE sensitization profiles and cross‐reactivity pattern were investigated in patients from three geographically distinct regions. Endolysosomal degradation assays were performed to monitor proteolytic susceptibility, and T‐cell assays were conducted to analyze cross‐reactivity at the T‐cell level.

## 2 METHODS

Detailed description of all experimental procedures is provided in the Data S1.

### 2.1 Patients and sera

Weed pollen‐allergic patients from Austria (n = 36), Canada (n = 38), and Korea (n = 24) were selected on the basis of typical case history, that is, rhinitis and/or conjunctivitis during late summer, and allergen‐specific IgE to mugwort and/or ragweed pollen determined by ImmunoCAP or skin prick test (Table S1). Experiments using anonymized serum samples from allergic patients were approved by the Ethics Committee of the Medical University of Vienna, Austria (No. 712/2010), the IRB—Institutional Review Board Services, Aurora, Ontario, Canada, and the Institutional Review Board of the Yonsei University Health System, Seoul, Korea (No. 4‐2013‐0397). Informed written consents were obtained from all individuals.

### 2.2 In silico analysis, protein expression, and purification

In silico analysis of the defensin‐like domain was performed with Clustal Omega, SWISS‐MODEL, and Chimera 1.8. Constructs corresponding to the mature sequences of Art v 1.0101, Amb a 4.0101, and Par h 1.0101 were cloned into the pHis Parallel 2 expression vector.^13^, ^15^ Protein expression of nontagged proteins was performed in *E. coli* Rosetta‐gamiB (DE3) pLysS as described.^15^ Briefly, Art v 1 was obtained by ultrafiltration followed by cation exchange and size exclusion chromatography. Amb a 4 and Par h 1 were purified using ammonium sulfate precipitation followed by hydrophobic interaction and size exclusion chromatography.

### 2.3 Physicochemical characterization

Purified allergens were analyzed by reducing SDS‐PAGE and Coomassie staining. Protein concentrations were determined by amino acid analysis, and identity was verified by intact mass analyses using a Q‐Exactive mass spectrometer. The aggregation behavior of the proteins was analyzed by dynamic light scattering (DLS) and high‐performance liquid chromatography (HPLC). Secondary structure elements were determined by Fourier transform infrared (FTIR) spectroscopy using a Tensor 27 spectrophotometer. Circular dichroism spectra of native and heat‐treated proteins were recorded with a JASCO J‐815 spectropolarimeter.

### 2.4 IgE reactivity, cross‐inhibition, and mediator release assay

IgE reactivity of weed pollen extracts and purified allergens was evaluated by ELISA using sera from weed pollen‐allergic patients. The biological activity of the allergens was verified in a mediator release assay.^21^ Detection of IgE reactivity to purified allergens was performed using a colorimetric ELISA. IgE reactivity to reduced/alkylated (R/A) allergens was assessed using a chemiluminescence ELISA. IgE cross‐reactivity between purified allergens was studied by cross‐inhibition ELISA. Sera were pre‐incubated overnight with the inhibitor molecules or buffer.

### 2.5 Proteolytic stability

The proteolytic stability was studied with the endolysosomal degradation assays.^22^ Recombinant allergens were incubated with the microsomal fraction of a murine dendritic cell (DC) line or purified recombinant cathepsin S. Samples were analyzed at different time points using gel electrophoresis and mass spectrometry.

### 2.6 T‐cell reactivity

Monocyte‐derived dendritic cells were differentiated as described^23^, ^24^ and incubated with the allergens or Art v 1‐peptide. Art v 1_25‐36_‐specific TCR tg Jurkat T‐cells^25^, ^26^ were added and cocultured and IL‐2 promoter‐driven luciferase activity was determined.^25^ Art v 1_25‐36_‐specific T‐cell lines and clones established from mugwort‐allergic donors were tested for proliferation in response to the allergens or Art v 1‐peptide by ^3^H‐thymidine uptake.^20^

### 2.7 Data processing and statistical analyses

An unsupervised cluster analysis was performed with ClustVis using IgE reactivity and cross‐inhibition data.^27^ Statistical analyses were performed in GraphPad Prism using a Friedman test for nonparametric analyses followed by a Dunn's multiple comparison and Spearman's correlation tests.

## 3 RESULTS

### 3.1 Homologous allergens present typical features of defensin‐polyproline–linked proteins

**Figure 1:**
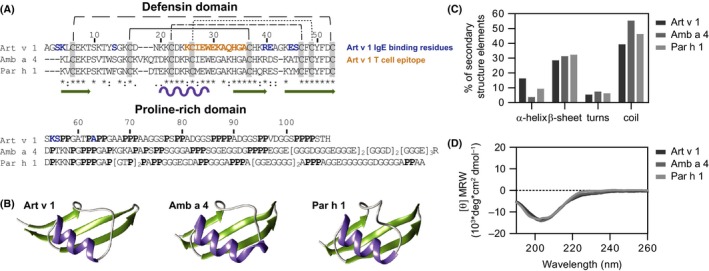
Art v 1, Amb a 4, and Par h 1 are homologous allergens with a defensin‐like fold. A, Sequence alignment of the defensin‐like domain and comparison of the proline‐rich region. β‐sheets and α‐helix from Art v 1 sequence are indicated by green arrows and purple waves, respectively. The lines above the sequence represent disulfide bonds. B, A ribbon side‐view representation of the three‐dimensional structure of Art v 1 (PDB: 2kpy) and structure models of Amb a 4 and Par h 1. C, Percentage of the secondary structure elements calculated from the FTIR measurements. D, Circular dichroism spectra of the recombinant proteins measured at 20°C

To investigate the physicochemical and structural features of Art v 1, Amb a 4, and Par h 1, the allergens were expressed as nontagged proteins in *E. coli*. They shared the prototypical features of defensin‐polyproline–linked proteins with an N‐terminal defensin‐like domain and eight conserved cysteine residues.^19^ Sequence identities ranging from 67% to 78% in the defensin‐like domain were noted (Figure 1A). A common feature in the C‐terminal region is the high proline content, while there are major differences in length and amino acid composition. The proline‐rich regions of Amb a 4 and Par h 1 are longer and contain substantially more glycine residues. The high content of acidic amino acids in this region renders Amb a 4 and Par h 1 acidic in contrast to Art v 1. The structural models generated using Art v 1 as template revealed partially conserved IgE‐binding epitopes of the homologs (Figure 1B, Fig. S1). The proline‐rich region could not be modeled due to low sequence similarity and high flexibility.^19^

All three allergens were purified to homogeneity with purities of >98% (Fig. S2A). In line with previous results, they showed an unusual electrophoretic behavior.^13^, ^14^, ^15^ The protein identity and formation of four disulfide bonds were unequivocally verified by mass spectrometry (Fig. S2B). Protein preparations were monomeric with a R_h_ ~2 nm as assessed by DLS, and in HPLC, they eluted as single peaks with comparable retention times and molecular weight (Fig. S2C,D). FTIR analysis revealed the presence of defined secondary structure elements. Consistent with the structure of Art v 1, the proteins showed a higher content of beta sheets than alpha helices, and the amount of disordered structures positively correlated with the length of the proline‐rich region (Figure 1C and Fig. S3A). In CD analyses, almost identical spectra were recorded, indicating a similar folding consistent with proline‐rich proteins (Figure 1D).^13^, ^15^, ^21^ The defensin‐polyproline–linked proteins exhibited comparable stability to thermal denaturation, and structural changes observed at 222 nm were analogous. However, denaturation spectra of Amb a 4 and Par h 1 shared higher similarity compared to Art v 1 (Fig. S3B).

### 3.2 Weed pollen‐allergic patients from different geographic regions demonstrate distinct sensitization profiles

**Figure 2:**
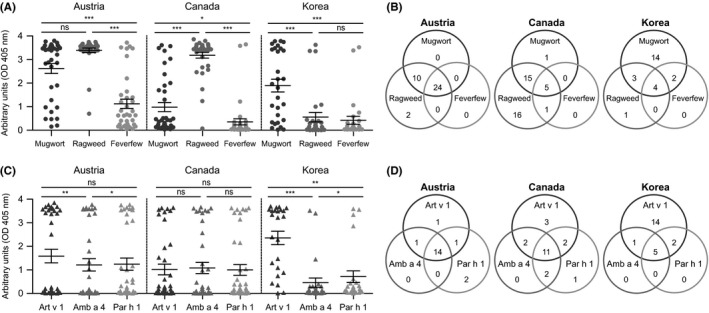
IgE sensitization prevalence investigated in patients’ sera from three geographic regions. IgE reactivity of patients’ sera to weed pollen extracts (A) and recombinant allergens (C). Sensitization profiles of (B) weed pollen extracts and (D) recombinant allergens depicted in Venn diagrams. Graphs show mean ± SEM, ***P ≤ .001, **P ≤ .01, *P ≤ .05 and ns, not significant

To assess the immunological relevance, we investigated sera of weed pollen‐allergic patients from Austria, Canada, and Korea. First, we evaluated the patients’ sensitization profile to pollen extracts of mugwort, ragweed, and feverfew in ELISA (Figure 2A,B). The majority of Austrian patients were multisensitized to all three sources, with ragweed presenting strongest IgE binding followed by mugwort pollen. While 53% of the Canadian patients reacted to ragweed as well as mugwort, a strong bias toward ragweed sensitization was noted which was also reflected by significantly higher IgE levels. The Korean patients showed a dominant mugwort sensitization profile with 58% mono‐sensitized patients.

The allergenic activity of all recombinant allergen preparations was verified in a mediator release assay (Fig. S4), and IgE reactivity of individual sera was monitored in ELISA (Figure 2C,D, Table S1). Sensitization to any of the tested molecules was detected in 19/36, 21/38, and 22/24 of the Austrian, Canadian, and Korean patients, respectively. Seventy‐four percent of patients from Austria and 52% from Canada who reacted to one of the tested allergens were also positive for the homologous molecules with similar IgE levels. Conversely, most Korean patients were exclusively sensitized to Art v 1, which is also reflected by significantly higher IgE levels. Of note, highly correlating IgE levels were observed between Amb a 4 and Par h 1, while correlations with Art v 1 were lower especially among Korean patients (Fig. S5).

### 3.3 Defensin‐polyproline–linked allergens demonstrate varying degrees of IgE cross‐reactivity

**Figure 3:**
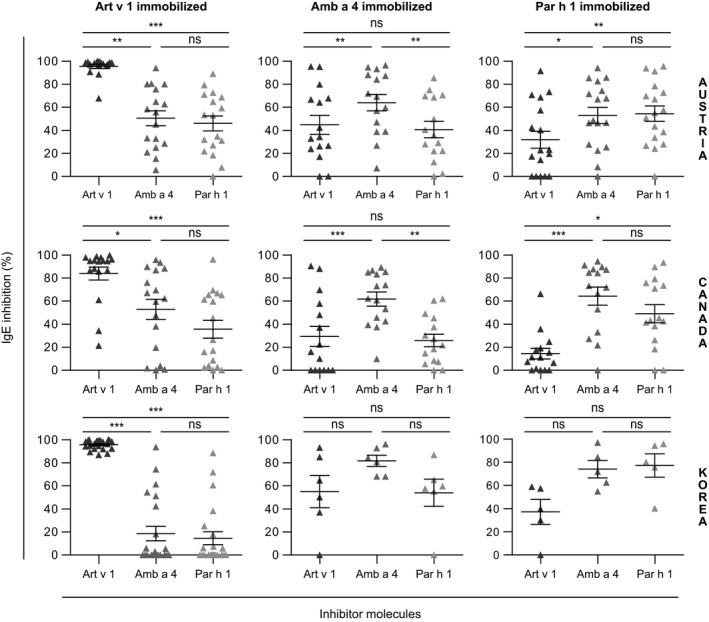
Defensin‐polyproline–linked allergens show variable IgE cross‐reactivity. Art v 1, Amb a 4, and Par h 1 were immobilized onto ELISA plates and patients’ sera were pre‐incubated with the inhibiting molecules. Graphs show mean ± SEM, ***P ≤ .001, **P ≤ .01, *P ≤ .05 and ns, not significant

In order to test the IgE cross‐reactivity, we performed an ELISA inhibition study with reactive patients’ sera (Figure 3). When Art v 1 was immobilized, Amb a 4 and Par h 1 were cross‐inhibiting with a mean of 50% and 46% in the Austrian patients, respectively. Among Canadian patients, similar results were observed although Amb a 4 was slightly more potent than Par h 1, but statistically not significant. Low cross‐inhibition potential was observed among Korean patients, where Amb a 4 and Par h 1 showed only 19% and 15% cross‐reactivity with Art v 1, respectively. Generally, sera from Austrian patients demonstrated broader IgE cross‐reactivity with Art v 1 in comparison with Korean patients where cross‐inhibition was restricted to only few patients. Inhibition to immobilized Amb a 4 ranged from 41% to 55% in the Austrian and Korean patients, while mean inhibition was <30% in Canadian patients’ sera. When Par h 1 was immobilized, we observed a similar inhibitory capacity of Amb a 4 and Par h 1, with Amb a 4 inhibition even more pronounced in some Canadian sera. Compared to Amb a 4, Art v 1 proved to be a weaker inhibitor for Par h 1 which was most prominent in the Canadian group.

### 3.4 IgE reactivity is differently affected upon structural alteration of the defensin‐like fold

**Figure 4:**
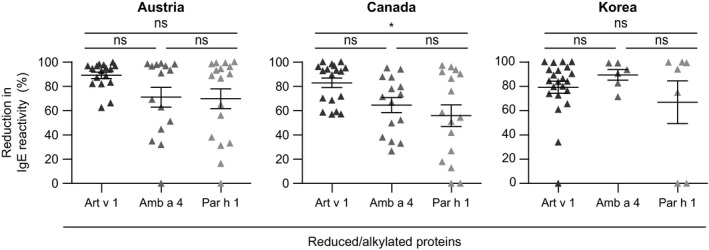
Amb a 4 and Par h 1 bear IgE epitopes which are not shared with Art v 1. IgE reactivity to native and reduced/alkylated allergens was evaluated in ELISA and percentage of reduction in IgE reactivity is depicted. Graphs show mean ± SEM, *P ≤ .05 and ns, not significant

IgE reactivity of Art v 1 is highly dependent on the cysteine‐stabilized defensin‐like fold, which prompted us to investigate the susceptibility of the homologous molecules.^13^, ^21^ Successful reduction/alkylation of proteins was verified by mass spectrometry and an increase of unordered structures was observed in CD and FTIR measurements (Fig. S6). For ELISA analyses, we used a chemiluminescence system in order to efficiently monitor changes in IgE binding. As expected, reduction/alkylation of the defensin‐like domain essentially abolished IgE binding to Art v 1 (Figure 4). Interestingly, IgE reactivity to reduced/alkylated Amb a 4 and Par h 1 showed a different profile as several Austrian and Canadian sera still recognized the structurally altered molecules. The majority of tested sera demonstrated loss of IgE reactivity, while others were still able to substantially bind to structurally altered Amb a 4 and Par h 1 with similar efficiency (Fig. S7). On the other hand, IgE reactivity to reduced/alkylated proteins was diminished for all allergens among Korean patients. An unsupervised clustering analysis using sera reactive to all three allergens revealed three patients’ groups (Fig. S8).

### 3.5 Defensin‐polyproline–linked allergens demonstrate different susceptibility to endolysosomal degradation

**Figure 5:**
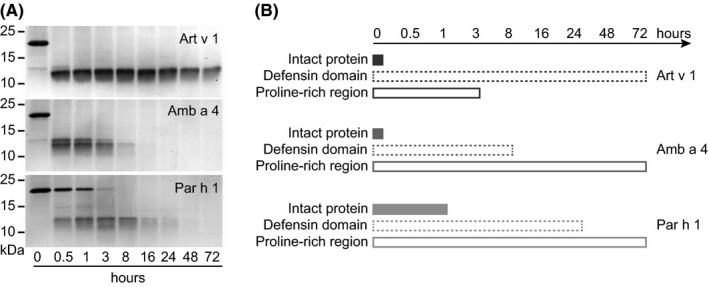
Defensin‐polyproline–linked allergens present different susceptibility to endolysosomal degradation using microsomal fraction of a murine DC line. A, Time‐dependent proteolytic degradation was monitored by SDS‐PAGE and Coomassie staining. B, Intact mass analysis was performed by mass spectrometry

**Figure 6:**
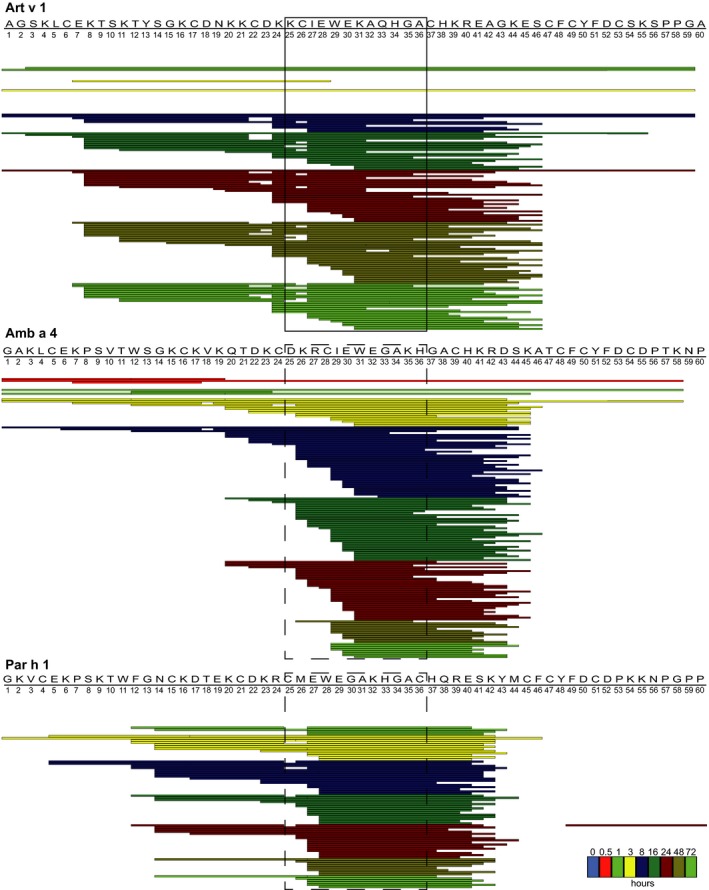
Endolysosomal degradation patterns within the defensin‐like domains. Peptide pattern was obtained after incubation of the allergens with endolysosomal proteins from a murine DC line and analyzed by mass spectrometry. The immunodominant T‐cell epitope Art v 1_25‐36_ is boxed with solid lines; homologous regions are boxed with dashed lines

Using the endolysosomal degradation assay, we analyzed kinetics and proteolytic degradation pattern by gel electrophoresis and mass spectrometry, respectively. Proteins were susceptible to cleavage of the intact protein within 0.5‐3 hours (Figure 5A). Degradation of the proteins yielded proteolytic products with clear differences in kinetics, with highest stability for a 12‐kDa Art v 1 fragment. All proteins underwent an early proteolytic cleavage resulting in separation of the two domains. Notably, the defensin‐like domain of Art v 1 was stable for 72 hours while those of Amb a 4 and Par h 1 were mostly degraded within 8 and 16 hours, respectively (Figure 5B). Major differences were observed regarding the proline‐rich region; the C‐terminal fragments of Amb a 4 and Par h 1 were detectable up to 72 hours while Art v 1 was highly susceptible to proteolysis (Figure 5B). Proteolytic peptides identified by mass spectrometry supported these findings (Fig. S9). Similar degradation patterns were found within the conserved defensin‐like domain although kinetics varied considerably (Figure 6). Typically, defensin‐like domains were degraded from both ends to the inner core and generated peptides were spanning the immunodominant T‐cell epitope of Art v 1.^20^, ^26^ Analogous degradation kinetics and cleavage sites were observed using cathepsin S (Fig. S10).

These results prompted us to investigate whether the homologous allergens were cross‐reactive at the T‐cell level. Therefore, we used Jurkat T‐cells transfected with a TCR specific for the immunodominant T‐cell epitope of Art v 1 as well as T‐cell lines and clones specific for Art v 1.^20^, ^25^ Under these experimental conditions, Amb a 4 and Par h 1 did neither demonstrate T‐cell cross‐reactivity with the TCR specific for Art v 1_25‐36_, nor with Art v 1‐specific T‐cells restricted to HLA‐DR1 (Fig. S11).

## 4 DISCUSSION

Relevant pollen allergens of the Asteraceae family belong to pectate lyases, defensin‐like domain linked to a polyproline region, and lipid transfer proteins.^2^, ^7^ With the exception of Art v 1, allergenic defensin‐polyproline–linked proteins are only scarcely investigated. While Art v 1 is considered the major allergen of mugwort pollen,^13^ homologs from ragweed and feverfew are considered minor allergens.^14^, ^15^ It is believed that distinct IgE sensitization profiles may arise from varying exposure to weeds in different geographic regions.^7^, ^9^ We further investigated whether intrinsic molecular characteristics of defensin‐polyproline–linked allergens could also account for distinct sensitization profiles.

Recombinant allergens showed typical structural features of the defensin‐like protein family while amino acid sequences vastly differed in the proline‐rich region.^13^, ^14^, ^15^ Structural modeling showed that IgE epitopes of Art v 1 are partially conserved in Amb a 4 and Par h 1. The proline‐rich region did not allow reliable prediction due to low sequence similarity and high structural flexibility.^19^, ^28^ In FTIR, comparable distributions of secondary structure elements were found which reflected previous NMR data obtained for Art v 1 in solution.^19^ Overall, proteins showed similar spectra in CD measurements and neither dimerization nor aggregation was identified for any of the protein batches. Thermal denaturation analyses showed similar unfolding pattern while melting curves of Amb a 4 and Par h 1 were different from Art v 1 suggesting divergent unfolding paths. Due to the high content of unordered motifs, interpretation of structural changes relating to the defensin‐like domain has to be made with caution. Tentatively, the flexible polyproline regions account for different physiological roles as a consequence of different amino acid content, net charge, and length.^28^, ^29^, ^30^

Next, the sensitization profile to weed pollen was investigated in patients from Austria, Canada, and Korea. Patients from Austria were sensitized to ragweed and mugwort due to the concomitant growth of both weeds in this region^7^, ^31^; only two patients were exclusively reactive to ragweed extract. The Canadian patients showed a prevalent sensitization to ragweed,^7^, ^31^ while 55% were also reactive to mugwort due to either IgE cosensitization or cross‐reactivity.^32^, ^33^ In contrast, Korean patients presented genuine sensitization to mugwort, which corresponded to the dominant Asteraceae weed sensitization in this region.^34^ IgE sensitization profiles to feverfew pollen were recorded in order to evaluate cross‐reactivity in Austrian and Canadian patients typically not exposed to this weed. Growth of feverfew has been reported in Southern Korea^4^ (http://www.cabi.org/isc/datasheet/45573); however, its implication in allergy has not been established.

Using recombinant allergens, a dominant IgE reactivity to Art v 1 (92%) among Korean patients was noted. Art v 1 sensitization prevalence in Canada and Austria was only 55% and 53%, respectively, suggesting that those patients were also reactive to Amb a 1.^7^, ^31^ Notably, Art v 1 and Par h 1 represent the most prevalent molecules in the pollen extract, while Amb a 4 levels are outperformed by the major allergen Amb a 1 (unpublished data). Among Austrian patients sensitized to defensin‐polyproline–linked proteins, 73% reacted to all three allergens in a similar manner with slightly higher IgE reactivity to Art v 1. The sensitization prevalence of 42% to Amb a 4 was in agreement with 39% as previously described.^14^ Comparable results were obtained for Par h 1 (47%) refining a 40% and 60% prevalence determined before in a limited group of Indian and Austrian patients, respectively.^15^ Two sera showed exclusive reactivity to Par h 1 which may be a consequence of exposure during traveling or living in countries where feverfew is endemic. Among Austrian patients, Art v 1 was more dominant at IgE cross‐inhibition, suggesting that most of the patients were primary sensitized to this mugwort allergen.^14^ Among Canadian patients who demonstrated particularly lower mugwort than ragweed pollen sensitization, similar but rather weak IgE levels to all molecules were observed. In this scenario, Art v 1 showed the same intensity of IgE reactivity as Amb a 4 and Par h 1, although the sensitization prevalence to Art v 1 was the same as in the Austrian group (47%). As patients in northern America are frequently sensitized to ragweed, we could speculate that Amb a 4 acts as primary sensitizer.^14^, ^33^ This is supported by inhibition ELISA results, with Amb a 4 being more efficiently inhibiting Art v 1 than vice versa. An analogous observation has been made for the pectate lyases Amb a 1 and Art v 6 corroborating that primary sensitization is highly dependent on weed pollen exposure.^33^, ^35^ In Korea, Art v 1 showed a dominant IgE reactivity, with only eight patients additionally reacting to the homologous allergens. This result might relate to strong affinity maturation of IgE antibodies due to continuous mugwort pollen exposure.^34^, ^36^ Notably, Amb a 4 and Par h 1 showed low inhibitory capacities in blocking IgE binding to Art v 1 in these patients. In all patients, IgE reactivity to Par h 1 was virtually the same as for Amb a 4, also reflected by highly correlating antibody levels. Even though Par h 1 sensitization has to be considered a cross‐reactivity phenomenon in the investigated context, this might have implications for patients sensitized to Art v 1 or Amb a 4.

To study reactivity pattern in detail, we investigated whether reduction and alkylation lead to loss of IgE reactivity. In line with previous studies, the appropriate folding of the Art v 1 defensin‐like domain was crucial for IgE reactivity.^13^, ^21^ However, when the folding of Amb a 4 or Par h 1 was altered, we observed a different trend among Austrian and Canadian patients. Interestingly, among those patients where IgE reactivity to Amb a 4 and Par h 1 was less affected by reduction and alkylation, Art v 1 was worse in inhibiting the IgE reactivity to these molecules. The observations were confirmed by unsupervised clustering analysis considering IgE reactivity, impairment due to allergen reduction and alkylation, as well as IgE cross‐reactivity of patients sensitized to all three allergens. Results suggest at least one cross‐reactive epitope located in the defensin‐like domain and in fact three amino acids of the IgE‐binding epitope of Art v 1 are conserved in the homologs.^19^ In this context, Amb a 4 and Par h 1 seem to possess an epitope which is not shared with Art v 1 and reflects either a linear epitope in the defensin‐like domain or is located in the proline‐rich region. Cluster analysis suggested that most of the patients where Art v 1 failed to inhibit IgE reactivity to Amb a 4 and Par h 1 were from Canada and thus primary sensitized to ragweed pollen. However, a considerable number of Austrian patients also demonstrated such a profile indicating primary sensitization to ragweed in this subgroup.

Several studies showed that protein immunogenicity is linked to endolysosomal processing during antigen presentation.^22^, ^37^, ^38^ All three proteins presented an early cleavage resulting in separation of the two domains. Subsequent degradation showed divergent profiles with high stability of the Art v 1 defensin‐like domain and proline‐rich region of Amb a 4 and Par h 1. The enhanced stability of the defensin‐like domain might explain the stronger immunogenicity of Art v 1, and in fact, the immunodominant T‐cell epitope is located in this region.^20^, ^39^ In contrast, premature degradation and thus suboptimal peptide loads resulting in poor presentation on MHC‐II might be a reason for a lower immunogenicity of Amb a 4 and potentially also Par h 1.^14^, ^37^, ^40^

As differences in the primary structure might provide distinct cleavage sites for different proteases, we additionally investigated the proteolytic stability to cathepsin S, a major component of the endolysosomal protease cocktail.^22^, ^41^, ^42^ Similar degradation kinetics was observed as compared with the endolysosomal extract and, in general, cathepsin S cleavage sites were conserved (Fig. S11). Potentially, other proteases of the endolysosomal fraction, varying intrinsic stability, or resistance to redox environment of proteins might account for minor differences.^43^, ^44^, ^45^ Proteolytic peptides typically harbored the immunodominant T‐cell epitope previously determined for Art v 1,^20^ which prompted us to investigate T‐cell cross‐reactivity. Despite high sequence similarity in this region, we observed no T‐cell cross‐reactivity using Art v 1‐specific T‐cell assays. Results are largely in agreement with Jahn‐Schmid et al^26^ who described that the crucial minimal Art v 1 T‐cell epitope (EWEKA) was restricted to HLA‐DR1. It is important to note that our experimental settings focused on the immunodominant epitope Art v 1_25‐36_, while T‐cell cross‐reactivity between other, minor epitopes and HLA class II restrictions other than those by HLA‐DR1 was not investigated.^20^, ^26^, ^39^ However, this finding also implicates that Art v 1 alone might not be efficient when considering molecule‐based immunotherapy approaches.

In summary, we show that regardless of the structural similarity shared by the allergenic defensin‐polyproline–linked proteins, they differ in their immunological properties. The higher sensitization rate of Art v 1 is consistent with the increased stability to proteolytic degradation, which ensures a steady supply of T‐cell epitopes. A group of patients reacted exclusively to Amb a 4/Par h 1 epitopes which are not dependent on the cysteine‐stabilized fold of the defensin‐like domain. We therefore suggest including Amb a 4 in molecule‐based diagnosis of weed pollen allergies, thus allowing further refinement for therapeutics in allergen immunotherapy.

## CONFLICTS OF INTEREST

Dr. Pablos Ocampo reports grants from Austrian Research Fund, during the conduct of the study; Dr. Eichhorn reports grants from Austrian Science Fund, during the conduct of the study; Dr. Machado reports grants from Austrian Science Fund, during the conduct of the study; Dr. Neunkirchner reports grants from Austrian Science Fund, during the conduct of the study; Dr. Jahn‐Schmid reports grants from Austrian Science Fund, grants from Austrian National Bank, during the conduct of the study; Dr. Wildner reports grants from Christian Doppler Research Association, during the conduct of the study; Dr. Pickl reports grants from Austrian Science Foundation (FWF), during the conduct of the study; and he holds stocks of Biomay AG, Vienna, Austria and received honoraria from Novartis for expert advise and seminars/talks; Dr. Arora reports grants from Department of Biotechnology, Government of India., during the conduct of the study; Prof. Vieths reports personal fees from Ärzteverband Deutscher Allergologen, personal fees from Swiss Society for Allergy and Immunology, personal fees from Schattauer Allergologie Handbuch, personal fees from Elsevier Nahrungsmittelallergien und Intoleranzen, personal fees from Karger Food Allergy: Molecular Basis and Clinical Practice, non‐financial support from German Research Foundation, non‐financial support from European Directorate for the Quality of Medicines and Health Care, non‐financial support from European Academy of Allergy and Clinical Immunology, non‐financial support from German Chemical Society (GDCh), non‐financial support from AKM Allergiekongress, non‐financial support from International Union of Immunological Societies, personal fees from University Hospital Gießen / Marburg, personal fees from University of Bonn, personal fees from Pharmacon, personal fees from Medical University of Vienna, Austria, personal fees from Gesellschaft für pädiatrische Allergologie und Umweltmedizin, non‐financial support from World Allergy Organization, non‐financial support from Technical University of Munich, non‐financial support from Austrian Society for Dermatology and Venerology, outside the submitted work; Dr. Gadermaier reports grants from Austrian Science Fund, during the conduct of the study; personal fees from Thermo Fisher Scientific, outside the submitted work. The other authors declare that they have no relevant conflict of interests.

## AUTHOR CONTRIBUTIONS

GG, FF, and SV were responsible for study concept and design. IP, SE, PB, NA, BJS, and SW performed experiments. IP, GG, FF, and YM analyzed and interpreted the data. WTS, CE, JWP, and WP provided reagents and materials. IP and GG drafted the manuscript. All authors provided critical revision of the manuscript for important intellectual content and approved the final version for publication.
