# Supplementary material for: Distinct epitope structures of defensin‐like proteins linked to proline‐rich regions give rise to differences in their allergenic activity
Source: Allergy. 2017 Sep 27;73(2):431–41. doi: 10.1111/all.13298 (PMC5771466; doi:10.1111/all.13298)
Supplement: Supplementary file 1 [file ALL-73-431-s001.docx]

**Distinct epitope structures of defensin-like proteins linked to proline rich regions give rise to differences in their allergenic activity**

Short title: Allergenic defensin-polyproline linked proteins

Isabel Pablos^1^, Stephanie Eichhorn^1^, Yoan Machado^1^, Peter Briza^1^, Alina Neunkirchner^2^, Beatrice Jahn-Schmid^3^, Sabrina Wildner^1,4^, Wai Tuck Soh^1^, Christof Ebner^5^, Jung-Won Park^6^, Winfried F. Pickl^2^, Naveen Arora^7^, Stefan Vieths^8^, Fatima Ferreira^1^, Gabriele Gadermaier^1^

^1^University of Salzburg, Department of Molecular Biology, Division of Allergy and Immunology, Salzburg, Austria

^2^Medical University of Vienna, Institute of Immunology, Center for Pathophysiology, Infectiology and Immunology, Vienna, Austria

^3^Medical University of Vienna, Department of Pathophysiology and Allergy Research, Vienna, Austria

^4^University of Salzburg, Christian Doppler Laboratory for Biosimilar Characterization, Salzburg, Austria

^5^Allergy Clinic Reumannplatz, Vienna, Austria

^6^Yonsei University College of Medicine, Department of Internal Medicine and Institute of Allergy, Seoul, Korea

^7^CSIR-Institute of Genomic and Integrative Biology, Allergy and Immunology Section, Delhi, India

^8^Paul-Ehrlich-Institut, Division of Allergology, Langen, Germany

**Corresponding author**

Gabriele Gadermaier

Department of Molecular Biology, University of Salzburg

Hellbrunnerstraße 34, A-5020 Salzburg, Austria

Telephone: 0043-662-8044-5974, Fax: 0043-662-8044-183

[Gabriele.Gadermaier@sbg.ac.at](mailto:Gabriele.Gadermaier@sbg.ac.at)


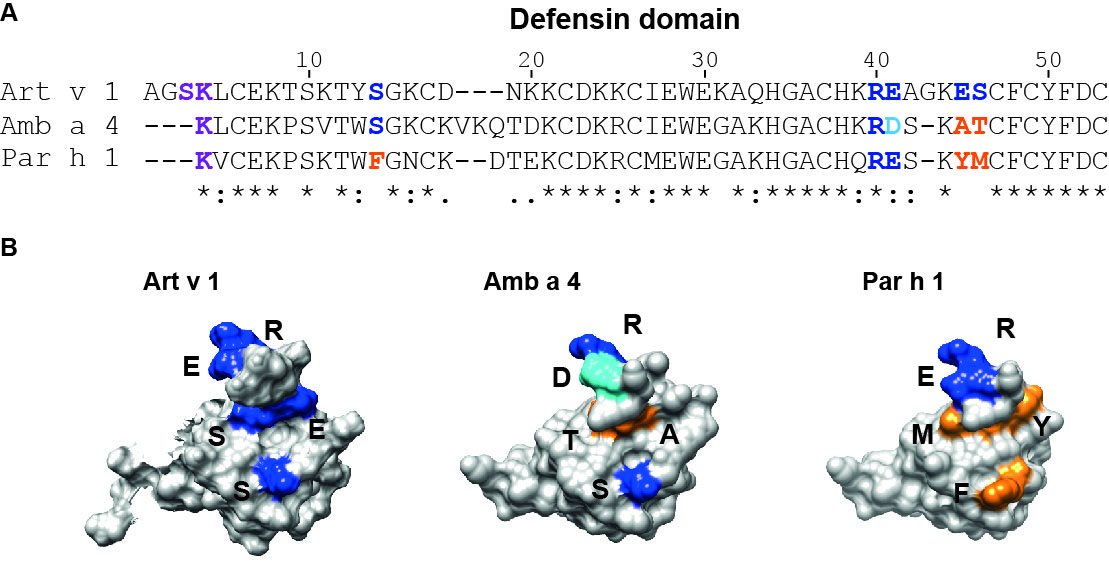


**FIG S1**. Conserved IgE-binding residues of Art v 1 and homologs. **A**, Sequence alignment of the defensin-like domain of Art v 1, Amb a 4 and Par h 1. Amino acids colored in pink and dark blue correspond to two IgE epitopes reported for Art v 1. Amino acids colored in pink or dark blue in Amb a 4 and Par h 1 are identical, colored in light blue conserved, colored in orange non-conserved substitutions. **B**, Surface top view representation of the three-dimensional structure of Art v 1 (PDB: 2kpy) and structure models of Amb a 4 and Par h 1. Colored regions on the surface structure correspond to the amino acids colored in the alignment.

**
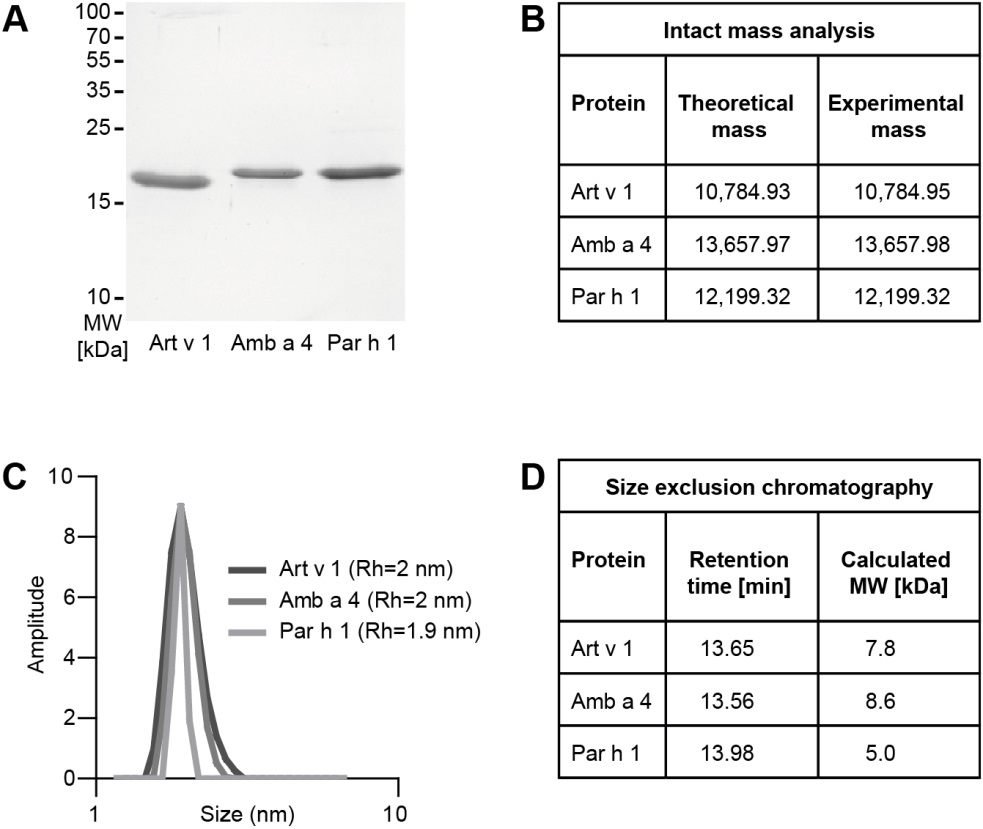
**

**FIG S2**. Defensin-polyproline linked allergens share physicochemical features. **A**, Reducing SDS-PAGE analysis and Coomassie staining of the purified allergens. **B**, Intact mass analysis of the purified allergens. **C**, Hydrodynamic radius (Rh) determined by dynamic light scattering (DLS). **D**, Retention times and calculated molecular weight obtained for each purified allergen as analyzed by size exclusion chromatography.


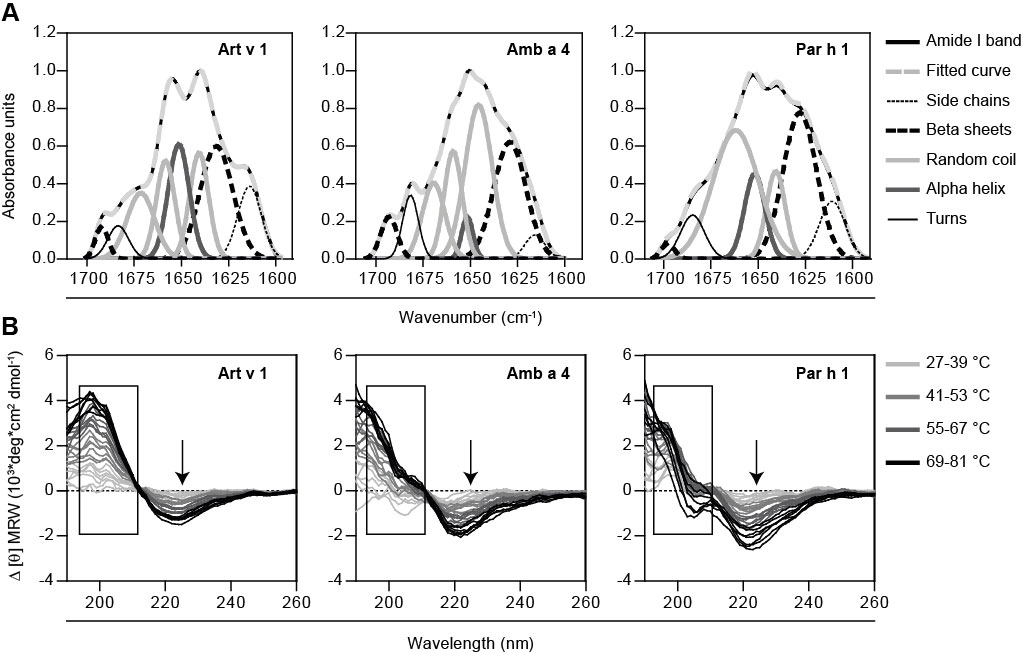


**FIG S3.** Defensin-polyproline linked allergens contain similar secondary structure elements and show comparable thermal stability. **A**, Absorbance spectra of the amide I deduced after Fourier self-deconvolution of the recombinant proteins. **B**, CD spectroscopy of defensin-polyproline linked proteins. Recombinant proteins were gradually heated from 25°C until 81°C and measurements were recorded every two degrees. To show the structural changes upon heating, the curves measured at 25°C were subtracted from the rest of the spectra. Regions of similar (arrows) and different changes (boxes) between the proteins are indicated.


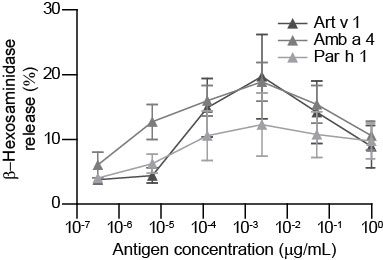


**FIG S4.** Defensin-polyproline linked allergens trigger degranulation of basophils. Rat basophil leukemia (RBL) cells transfected with the human IgE high affinity receptor (RBL-2H3) were sensitized with patients’ sera (n=7). The cells were stimulated with increasing concentration of recombinant allergens and the release of β-hexosaminidase was measured. The maximum release was obtained when the cells were treated with 10% of Triton X-100.


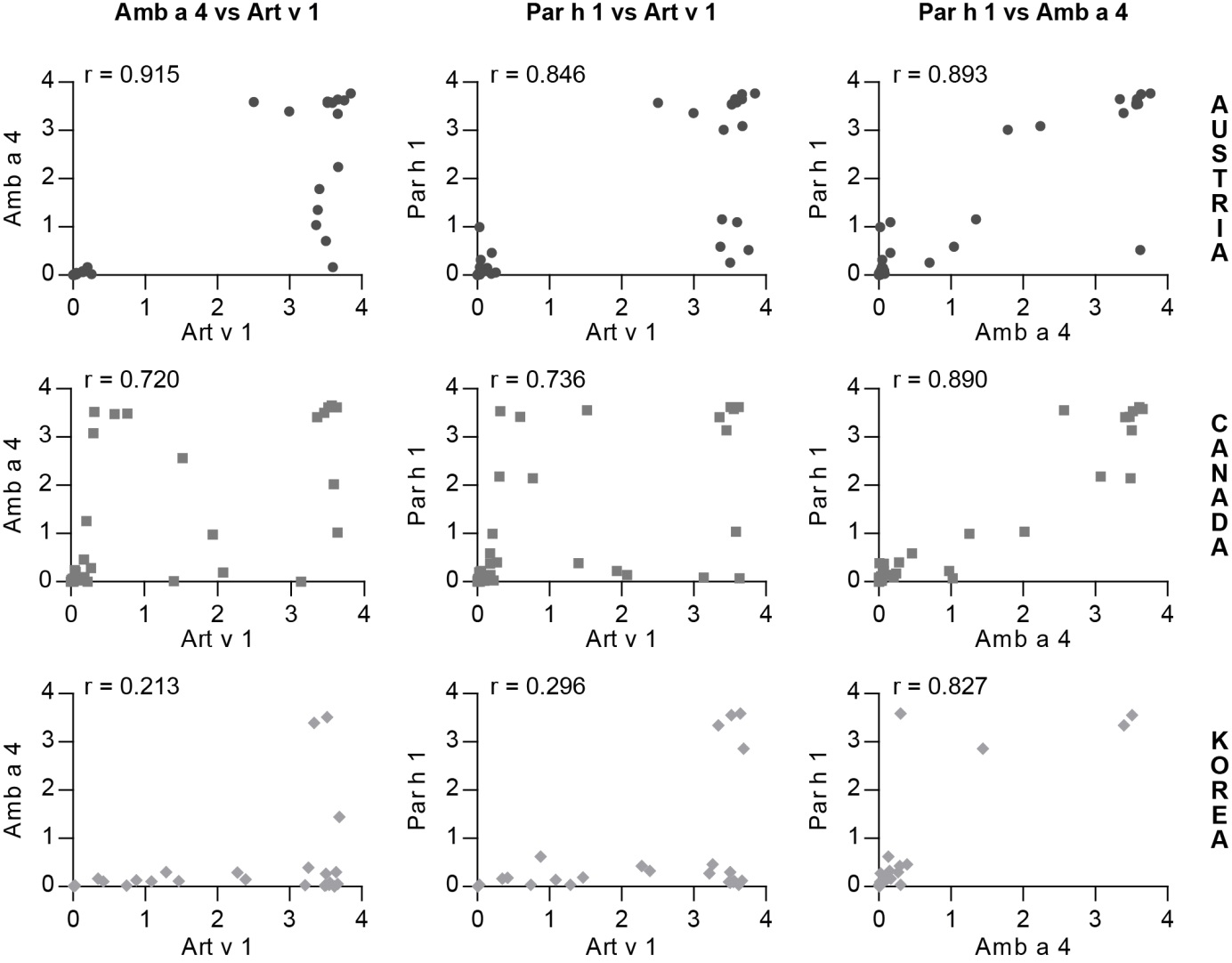


**FIG S5.** Correlation analyses of IgE reactivity to defensin-polyproline linked allergens. IgE reactivity of patients’ sera to recombinant allergens was measured by ELISA. Correlation analyses were performed in GraphPad Prism. The Spearman coefficient, r, is depicted.


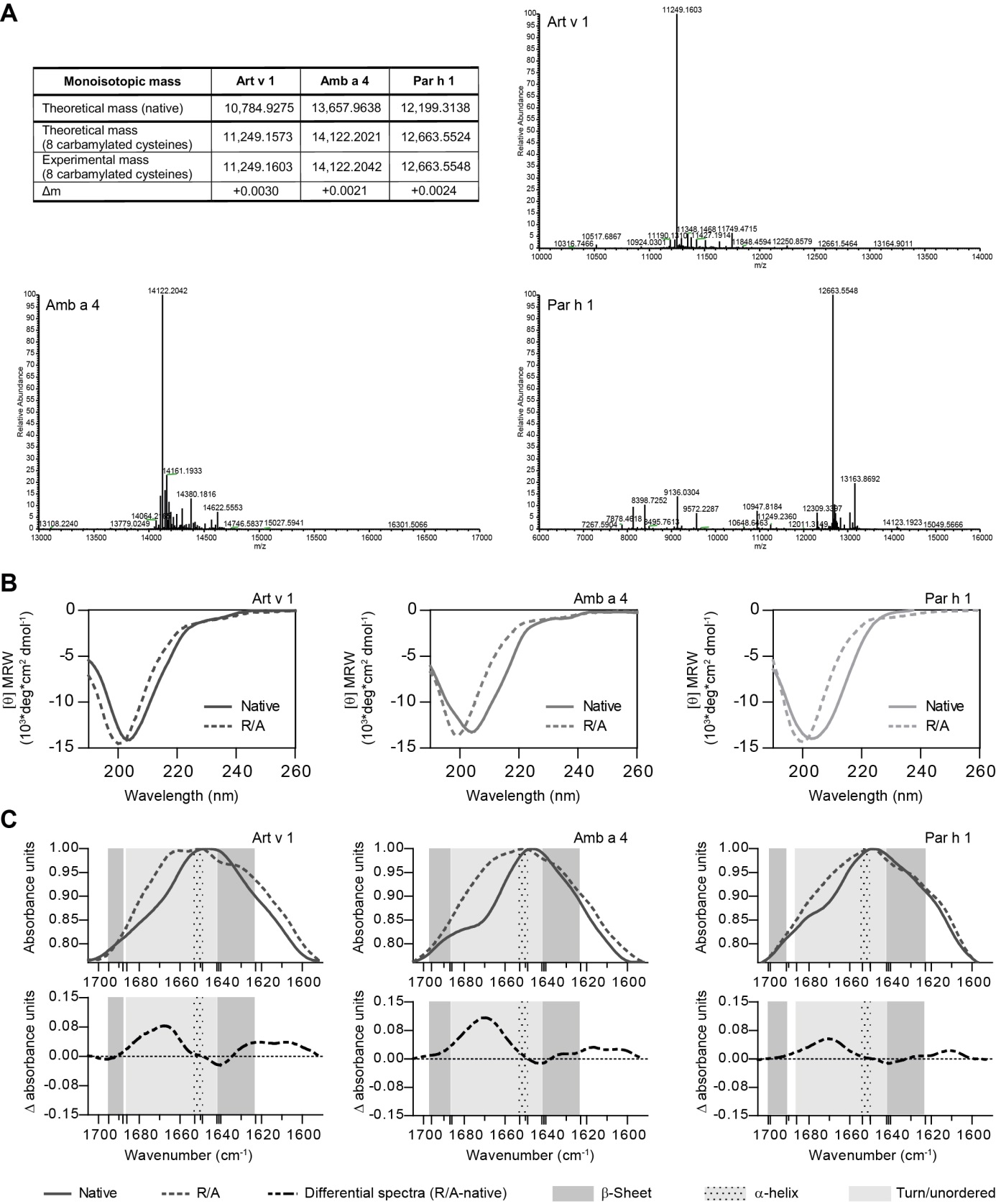


**FIG S6.** Defensin-polyproline linked allergens alter their secondary structure upon reduction and alkylation of the cysteines present in the defensin domain. **A**, Carbamylation of cysteine residues was confirmed by mass spectrometry analysis. Secondary structure analyses were performed using **B,** circular dichroism (CD) and **C,** Fourier transform infrared spectroscopy (FTIR). Native and reduced/alkylated (R/A) CD spectra were recorded at 20°C. Absorbance spectra of the amide I of native and R/A allergens are shown in the upper panel while differential spectra are shown in the lower panel. Shadowed regions correspond to the absorbance of secondary structure elements determined by FITR for each protein (Fig S3).


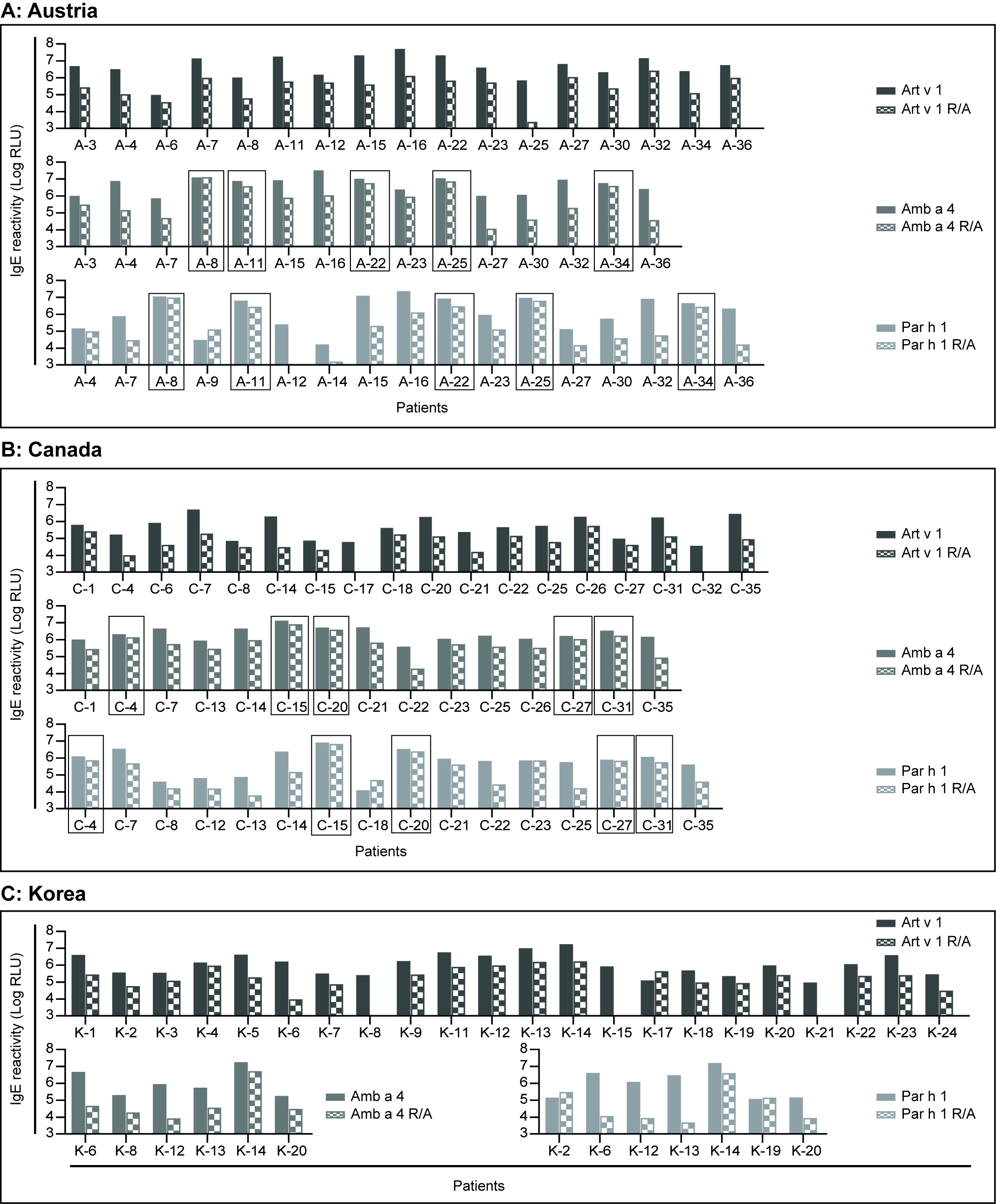


**FIG S7.** IgE reactivity to Amb a 4 and Par h 1 is differently affected by reduction/ alkylation of the defensin-like domain compared to Art v 1. Recombinant Art v 1, Amb a 4 and Par h 1 were reduced/ alkylated, immobilized onto the ELISA plates and the IgE reactivity was measured. The graphs depict the IgE reactivity of individual patients to proteins with intact defensin-like domain and structurally altered defensin-like domain (R/A). Patients’ sera where the IgE reactivity to R/A Amb a 4 and Par h 1 was not affected are enclosed in boxes.


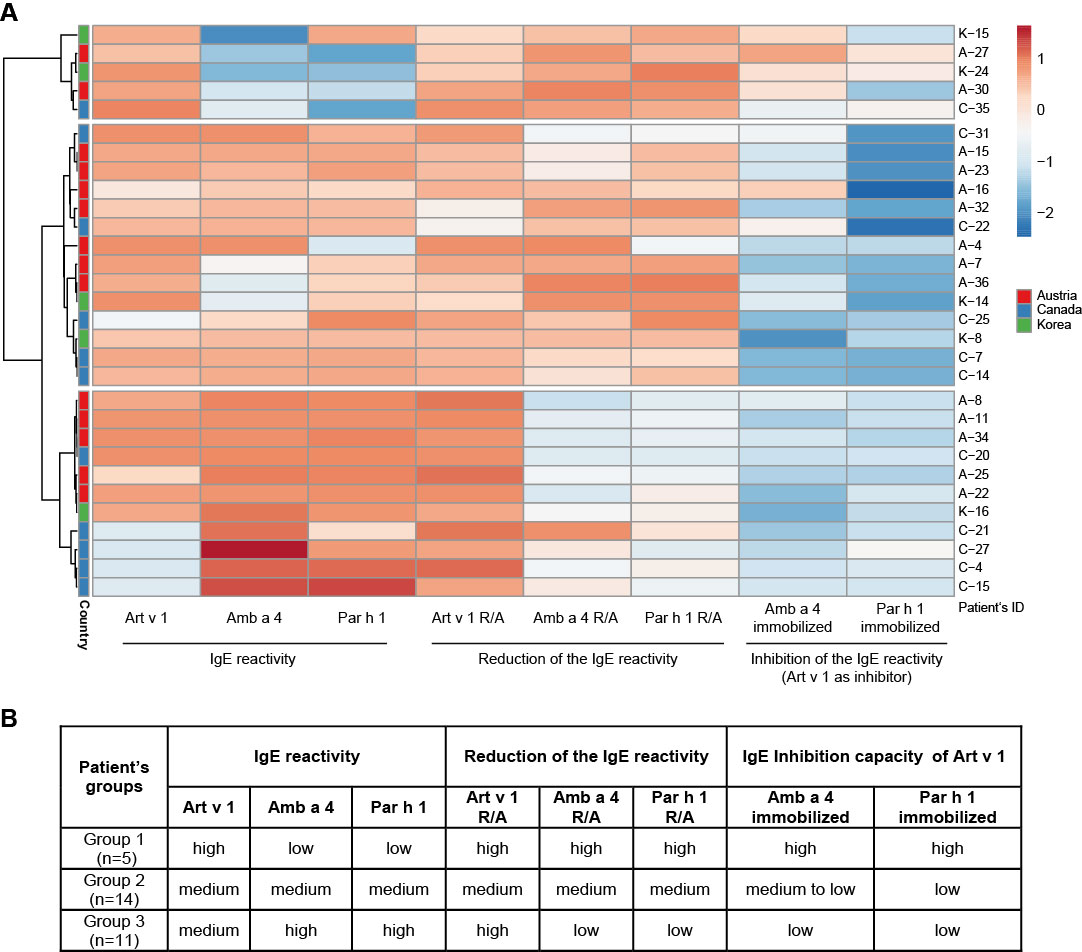


**FIG S8.** Amb a 4 and Par h 1 harbor IgE epitopes which are not shared with Art v 1. **A,** Cluster analysis performed with the online software ClustVis using the IgE reactivity to the defensin-polyproline linked allergens, the IgE reativity to the reduced/ alkylated allergens (R/A) and the IgE inhibition using Art v 1 as inhibitor molecule. **B,** Summarized results of the three groups identified by cluster analysis.


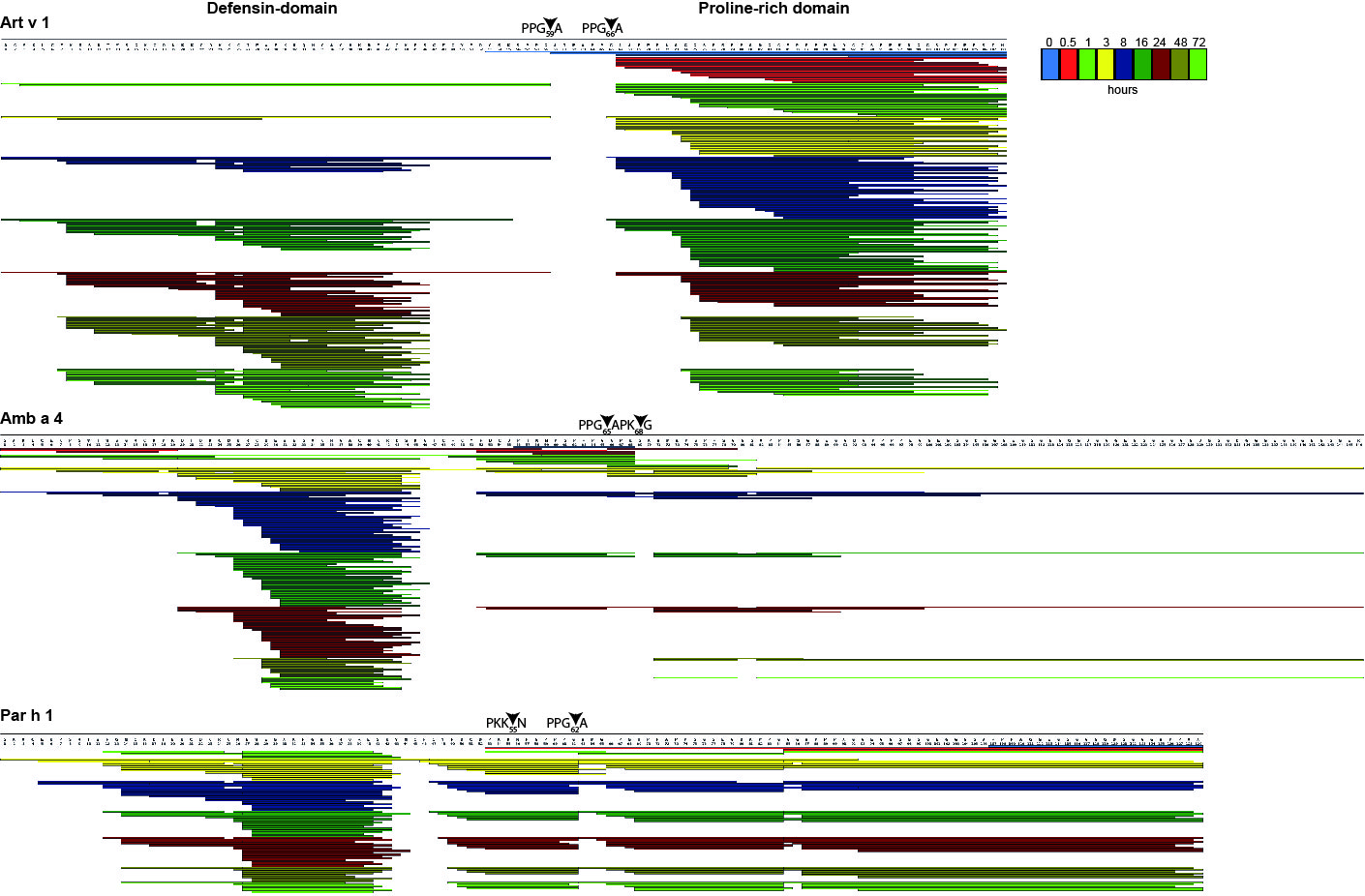


**FIG S9.** The defensin-like and proline rich domains showed different susceptibility to proteolytic degradation. Art v 1, Amb a 4 and Par h 1 were incubated with the microsomal fraction isolated from the mouse dendritic cell line JAWS II. The peptides generated in each time point were identified by mass spectrometry. Arrows indicate the amino acids of initial cleavage and separation of defensin-like and proline rich domain.


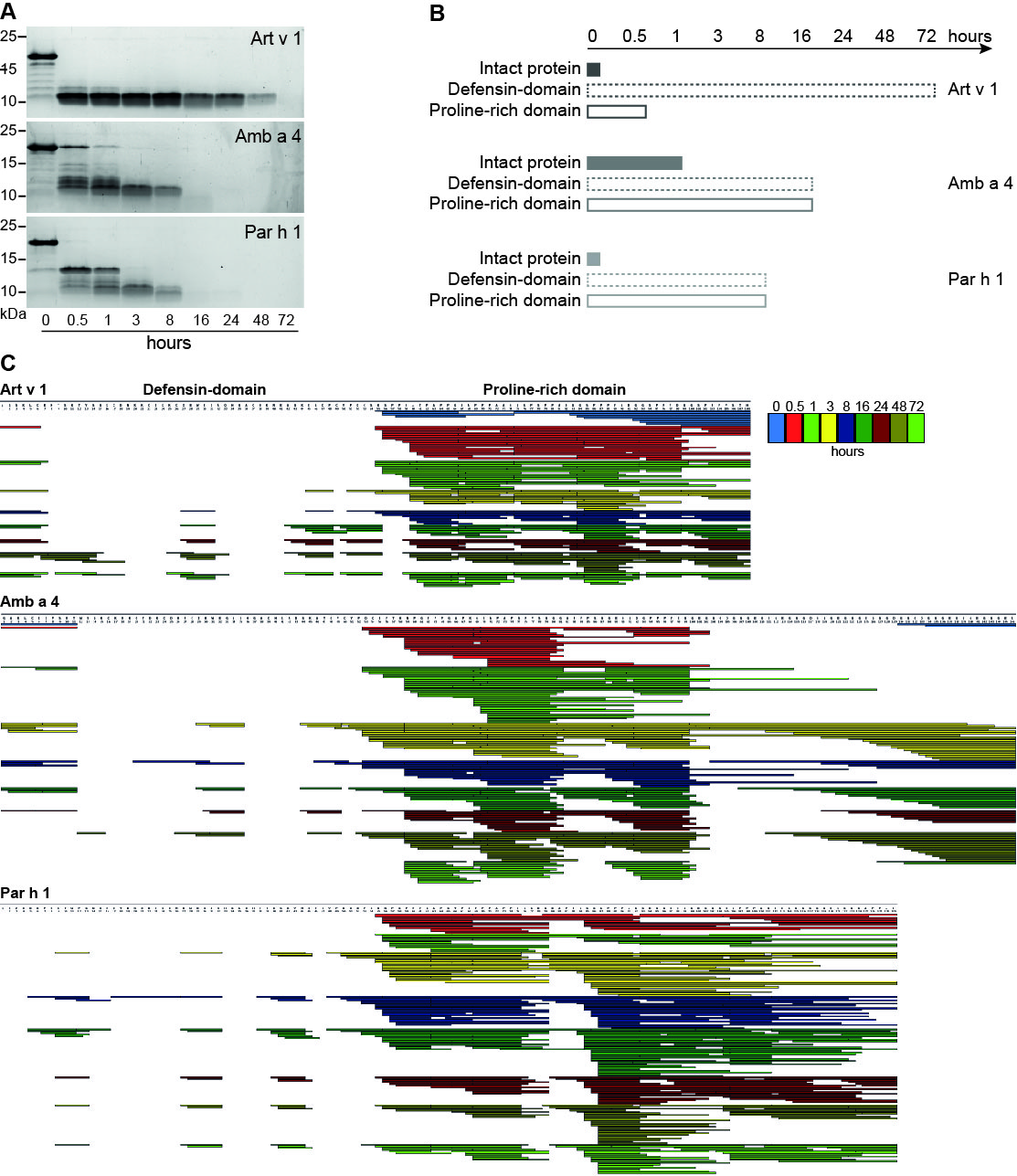


**FIG S10.** Degradation of defensin-polyproline linked allergens by cathepsins S reproduced the proteolytic behavior obtained by endolysosomal degradation. Art v 1, Amb a 4 and Par h 1 were incubated with pure cathepsin S. **A**, Time dependent proteolytic degradation was monitored by SDS-PAGE. **B**, Intact mass analysis was performed by mass spectrometry. **C**, The peptides generated in each time point were identified by mass spectrometry.


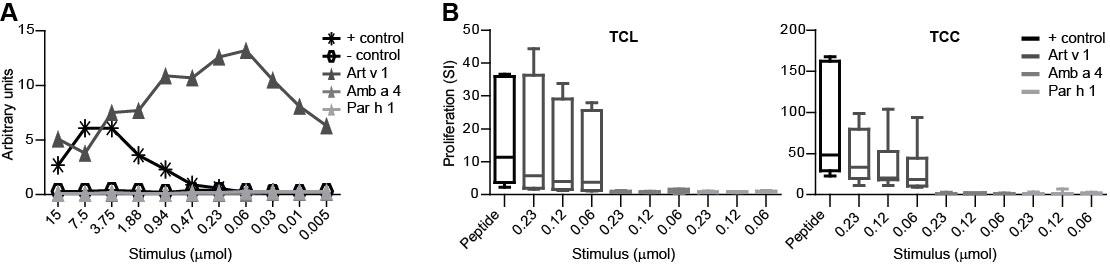


**FIG S11.** Amb a 4 and Par h 1 do not cross-react with the immunodominant T cell epitope of Art v 1 restricted to HLA-DR1. **A**, Proliferation of Jurkat T cells (TCR transgenic and DR1-restricted), specific for the immunodominant T cell epitope of Art v 1_25-36_. Art v 1 peptide K25-A36 (+ control) and recombinant Bet v 1 (- control). **B**, Proliferation of Art v 1 specific T cell lines (TCL; n=8 from 8 different subjects) and Art v 1 specific T cell clones (TCC; n=7 from 2 different subjects) measured by the incorporation of [3H] thymidine.
