# Supplementary material for: Distinct epitope structures of defensin‐like proteins linked to proline‐rich regions give rise to differences in their allergenic activity
Source: Allergy. 2017 Sep 27;73(2):431–41. doi: 10.1111/all.13298 (PMC5771466; doi:10.1111/all.13298)
Supplement: Supplementary file 2 [file ALL-73-431-s002.docx]

| **Austrian patients** | | | **ImmunoCAP**  **(RAST rating)** | | **IgE ELISA (arbitrary units – OD_405nm_)** | | | | | | **Experiments**  **performed** |
| --- | --- | --- | --- | --- | --- | --- | --- | --- | --- | --- | --- |
| **P-ID** | **Gender** | **Age** | **M** | **R** | **M** | **R** | **F** | **Art v 1** | **Amb a 4** | **Par h 1** |  |
| A-1 | m | 15 | 4 | 5 | 1.28 | 3.66 | 0.39 | 0.20 | 0.08 | 0.03 |  |
| A-2 | m | 46 | 3 | 4 | 2.88 | 3.47 | 1.39 | 0.04 | 0.02 | 0.03 |  |
| A-3 | m | 14 | 4 | 5 | 3.47 | 3.50 | 3.47 | 3.50 | 0.71 | 0.26 | I, R/A |
| A-4 | f | 41 | 3 | 5 | 3.60 | 3.56 | 0.67 | 3.76 | 3.62 | 0.52 | I, R/A |
| A-5 | m | 54 | 2 | 5 | 0.48 | 3.75 | 0.26 | 0.00 | 0.00 | 0.01 |  |
| A-6 | m | 22 | 3 | 5 | 2.86 | 3.48 | 0.12 | 0.26 | 0.02 | 0.06 | I, R/A |
| A-7 | f | 66 | 6 | 5 | 3.76 | 3.61 | 1.20 | 3.68 | 2.24 | 3.09 | I, R/A |
| A-8 | m | 60 | 3 | 5 | 3.42 | 3.36 | 0.16 | 3.00 | 3.39 | 3.36 | I, R/A |
| A-9 | m | 24 | 3 | 6 | 0.15 | 3.39 | 0.16 | 0.03 | 0.02 | 1.00 | I, R/A |
| A-10 | m | 12 | 5 | 6 | 3.44 | 3.45 | 0.63 | 0.05 | 0.03 | 0.10 |  |
| A-11 | m | 46 | 4 | 5 | 3.49 | 3.52 | 3.15 | 3.60 | 3.58 | 3.58 | I, R/A |
| A-12 | m | 19 | 5 | 5 | 3.64 | 1.72 | 1.37 | 3.60 | 0.16 | 1.10 | I, R/A |
| A-13 | m | 21 | 3 | 5 | 0.97 | 3.42 | 0.16 | 0.06 | 0.03 | 0.05 |  |
| A-14 | m | 15 | 3 | 4 | 2.99 | 3.50 | 0.62 | 0.20 | 0.16 | 0.46 | I, R/A |
| A-15 | f | 58 | 5 | 5 | 3.80 | 3.75 | 3.72 | 3.85 | 3.77 | 3.77 | I, R/A, RBL |
| A-16 | m | 10 | 5 | 5 | 3.56 | 3.52 | 3.52 | 3.53 | 3.60 | 3.54 | I, R/A |
| A-17 | f | 79 | 5 | 5 | 3.41 | 3.34 | 0.40 | 0.02 | 0.01 | 0.02 |  |
| A-18 | m | 29 | 4 | 6 | 2.70 | 3.35 | 1.50 | 0.04 | 0.02 | 0.04 |  |
| A-19 | m | 38 | 3 | 5 | 2.83 | 3.51 | 0.83 | 0.00 | 0.00 | 0.00 |  |
| A-20 | f | 33 | 4 | 5 | 3.21 | 3.55 | 1.64 | 0.13 | 0.08 | 0.09 |  |
| A-21 | f | 8 | n.d. | 5 | 1.82 | 3.56 | 1.01 | 0.05 | 0.05 | 0.32 |  |
| A-22 | m | 36 | 6 | 6 | 3.58 | 3.58 | 2.20 | 3.57 | 3.58 | 3.65 | I, R/A, RBL |
| A-23 | f | 44 | n.d. | 5 | 3.71 | 3.67 | 0.42 | 3.67 | 3.34 | 3.65 | I, R/A |
| A-24 | m | 41 | 3 | 5 | 0.97 | 3.53 | 0.06 | 0.04 | 0.03 | 0.02 |  |
| A-25 | f | 25 | 3 | 5 | 0.47 | 3.55 | 0.24 | 2.51 | 3.59 | 3.58 | I, R/A, RBL |
| A-26 | f | 71 | 4 | 5 | 0.20 | 3.28 | 0.05 | 0.02 | 0.01 | 0.04 |  |
| A-27 | m | 13 | 4 | 5 | 3.47 | 0.70 | 0.63 | 3.37 | 1.04 | 0.59 | I, R/A |
| A-28 | f | 38 | n.d. | 5 | 0.48 | 3.50 | 0.10 | 0.01 | 0.02 | 0.00 |  |
| A-29 | m | 12 | 5 | 6 | 3.49 | 3.53 | 3.56 | 0.04 | 0.04 | 0.17 |  |
| A-30 | f | 41 | 3 | 5 | 3.38 | 3.38 | 0.18 | 3.39 | 1.35 | 1.16 | I, R/A |
| A-31 | f | 51 | 5 | 5 | 3.59 | 3.57 | 1.22 | 0.04 | 0.03 | 0.06 |  |
| A-32 | m | 11 | 4 | 5 | 3.58 | 3.63 | 3.47 | 3.53 | 3.57 | 3.53 | I, R/A |
| A-33 | m | 28 | 2 | 5 | 0.78 | 3.66 | 0.24 | 0.05 | 0.03 | 0.09 |  |
| A-34 | f | 18 | 3 | 5 | 3.51 | 3.67 | 0.20 | 3.67 | 3.64 | 3.75 | I, R/A |
| A-35 | m | 16 | n.d. | 6 | 1.91 | 3.49 | 0.63 | 0.14 | 0.06 | 0.15 |  |
| A-36 | f | 49 | 4 | 3 | 3.49 | 3.42 | 0.81 | 3.41 | 1.79 | 3.02 | I, R/A, RBL |
|  |  |  |  |  | **94%** | **100%** | **67%** | **47%** | **42%** | **47%** |  |

**Online Table 1A**

| **Canadian Patients** | **ImmunoCAP**  **(RAST rating)** | | **IgE ELISA (arbitrary units – OD_405nm_)** | | | | | | **Experiments**  **performed** |
| --- | --- | --- | --- | --- | --- | --- | --- | --- | --- |
| **P-ID** | **M** | **R** | **M** | **R** | **F** | **Art v 1** | **Amb a 4** | **Par h 1** |  |
| C-1 | n.d. | 3 | 1.64 | 0.07 | 0.08 | 1.94 | 0.98 | 0.22 | I, R/A |
| C-2 | n.d. | 4 | 0.52 | 3.55 | 0.22 | 0.11 | 0.03 | 0.11 |  |
| C-3 | n.d. | 4 | 0.41 | 3.46 | 0.13 | 0.03 | 0.07 | 0.20 |  |
| C-4 | n.d. | 4 | 0.43 | 3.48 | 0.06 | 0.60 | 3.48 | 3.42 | I, R/A |
| C-5 | n.d. | 4 | 0.15 | 3.46 | 0.03 | 0.02 | 0.01 | 0.01 |  |
| C-6 | n.d. | 3 | 1.30 | 2.67 | 0.25 | 2.08 | 0.19 | 0.14 | I, R/A |
| C-7 | n.d. | 6 | 3.61 | 3.71 | 3.65 | 3.62 | 3.62 | 3.63 | I, R/A, RBL |
| C-8 | n.d. | 5 | 0.25 | 3.50 | 0.38 | 0.28 | 0.28 | 0.40 | I, R/A |
| C-9 | n.d. | 4 | 0.10 | 3.42 | 0.06 | 0.01 | 0.04 | 0.06 |  |
| C-10 | n.d. | 5 | 0.27 | 3.48 | 0.09 | 0.10 | 0.04 | 0.03 |  |
| C-11 | n.d. | 3 | 0.47 | 2.74 | 0.05 | 0.00 | 0.00 | 0.00 |  |
| C-12 | n.d. | 4 | 0.36 | 3.50 | 0.58 | 0.18 | 0.07 | 0.38 | I, R/A |
| C-13 | n.d. | 5 | 0.31 | 3.39 | 0.30 | 0.18 | 0.46 | 0.59 | I, R/A |
| C-14 | n.d. | 5 | 2.01 | 3.52 | 0.21 | 3.51 | 3.61 | 3.63 | I, R/A, RBL |
| C-15 | n.d. | 5 | 0.51 | 3.49 | 0.22 | 0.32 | 3.52 | 3.54 | I, R/A, RBL |
| C-16 | n.d. | 4 | 0.20 | 3.79 | 0.04 | 0.02 | 0.00 | 0.06 |  |
| C-17 | n.d. | 4 | 0.49 | 3.86 | 0.10 | 0.23 | 0.01 | 0.03 | I, R/A |
| C-18 | n.d. | 4 | 0.23 | 3.56 | 0.02 | 1.41 | 0.01 | 0.38 | I, R/A |
| C-19 | n.d. | 4 | 0.13 | 3.46 | 0.10 | 0.04 | 0.05 | 0.07 |  |
| C-20 | n.d. | 4 | 3.37 | 3.36 | 0.35 | 3.36 | 3.42 | 3.41 | I, R/A |
| C-21 | n.d. | 4 | 0.14 | 3.39 | 0.16 | 0.77 | 3.49 | 2.15 | I, R/A |
| C-22 | n.d. | 3 | 3.57 | 3.56 | 3.58 | 3.56 | 3.66 | 3.58 | I, R/A |
| C-23 | n.d. | 3 | 0.15 | 2.46 | 0.17 | 0.21 | 1.26 | 0.99 | I, R/A |
| C-24 | n.d. | 4 | 0.16 | 3.65 | 0.17 | 0.05 | 0.07 | 0.22 |  |
| C-25 | n.d. | 3 | 3.04 | 3.44 | 1.22 | 1.52 | 2.57 | 3.56 | I, R/A |
| C-26 | n.d. | 3 | 3.49 | 3.61 | 0.21 | 3.63 | 1.02 | 0.07 | I, R/A |
| C-27 | n.d. | 5 | 1.89 | 3.64 | 0.12 | 0.31 | 3.08 | 2.18 | I, R/A |
| C-28 | n.d. | 3 | 0.08 | 2.42 | 0.03 | 0.01 | 0.02 | 0.03 |  |
| C-29 | n.d. | 3 | 0.20 | 3.32 | 0.04 | 0.04 | 0.01 | 0.00 |  |
| C-30 | n.d. | 3 | 0.12 | 2.57 | 0.10 | 0.07 | 0.20 | 0.10 |  |
| C-31 | n.d. | 4 | 2.38 | 3.44 | 0.08 | 3.46 | 3.51 | 3.14 | I, R/A |
| C-32 | n.d. | 3 | 1.70 | 3.47 | 0.03 | 3.14 | 0.00 | 0.09 | I, R/A |
| C-33 | n.d. | 3 | 0.05 | 1.74 | 0.03 | 0.01 | 0.01 | 0.02 |  |
| C-34 | n.d. | 3 | 0.44 | 3.41 | 0.24 | 0.04 | 0.04 | 0.08 |  |
| C-35 | n.d. | 3 | 2.86 | 3.62 | 0.18 | 3.59 | 2.02 | 1.04 | I, R/A |
| C-36 | n.d. | 4 | 0.18 | 1.24 | 0.14 | 0.06 | 0.24 | 0.18 |  |
| C-37 | n.d. | 4 | 0.03 | 3.11 | 0.00 | 0.00 | 0.01 | 0.02 |  |
| C-38 | n.d. | 3 | 0.13 | 3.50 | 0.15 | 0.19 | 0.10 | 0.14 |  |
|  |  |  | **53%** | **97%** | **16%** | **47%** | **39%** | **42%** |  |

**Online Table 1B**

**Online Table 1C**

| **Korean Patients** | | | **ImmunoCAP**  **(RAST rating)** | | **SPT** | | **IgE ELISA (arbitrary units – OD_405nm_)** | | | | | | **Experiments**  **performed** |
| --- | --- | --- | --- | --- | --- | --- | --- | --- | --- | --- | --- | --- | --- |
| **P-ID** | **Gender** | **Age** | **M** | **R** | **M** | **R** | **M** | **R** | **F** | **Art v 1** | **Amb a 4** | **Par h 1** |  |
| K-1 | f | 30 | 3 | 0 | 12 | 6 | 3.43 | 0.02 | 0.04 | 3.49 | 0.02 | 0.10 | I, R/A |
| K-2 | m | 63 | 2 | 0 | 9 | 2 | 1.80 | 0.44 | 0.58 | 2.28 | 0.29 | 0.43 | I, R/A |
| K-3 | f | 36 | 3 | n.d. | 7 | 2 | 0.64 | 0.01 | 0.04 | 0.74 | 0.02 | 0.04 | I, R/A |
| K-4 | m | 34 | 3 | n.d. | 20 | 2 | 3.48 | 0.10 | 0.16 | 3.55 | 0.04 | 0.10 | I, R/A |
| K-5 | m | 29 | 3 | n.d. | 33 | 4 | 3.75 | 0.01 | 0.03 | 3.67 | 0.06 | 0.12 | I, R/A |
| K-6 | f | 60 | 2 | n.d. | 2 | 2 | 1.10 | 3.37 | 0.04 | 3.52 | 3.51 | 3.55 | I, R/A |
| K-7 | m | 38 | n.d. | n.d. | 12 | 4 | 0.43 | 0.17 | 0.17 | 0.42 | 0.10 | 0.18 | I, R/A |
| K-8 | m | 57 | n.d. | n.d. | 11 | 4 | 0.34 | 0.03 | 0.03 | 1.29 | 0.30 | 0.04 | I, R/A |
| K-9 | f | 35 | 3 | n.d. | 10 | 3 | 3.34 | 0.00 | 0.02 | 3.62 | 0.00 | 0.03 | I, R/A |
| K-10 | m | 59 | 3 | 0 | n.d. | n.d. | 1.96 | 0.04 | 0.07 | 0.03 | 0.02 | 0.04 |  |
| K-11 | f | 66 | 3 | 3 | n.d. | n.d. | 3.67 | 0.28 | 1.06 | 3.56 | 0.08 | 0.12 | I, R/A |
| K-12 | f | 56 | 3 | 2 | 20 | 18 | 3.78 | 3.62 | 3.52 | 3.69 | 1.45 | 2.86 | I, R/A |
| K-13 | f | 58 | 4 | 3 | 28 | 11 | 3.79 | 1.08 | 0.75 | 3.65 | 0.31 | 3.59 | I, R/A |
| K-14 | f | 44 | 3 | 1 | n.d. | n.d. | 3.47 | 3.36 | 3.39 | 3.34 | 3.40 | 3.34 | I, R/A |
| K-15 | f | 42 | 3 | 2 | 22 | 2 | 2.18 | 0.06 | 0.05 | 2.39 | 0.15 | 0.32 | I, R/A |
| K-16 | m | 75 | 0 | 3 | 13 | 8 | 0.02 | 1.00 | 0.04 | 0.01 | 0.01 | 0.00 |  |
| K-17 | m | 49 | 3 | n.d. | 13 | 2 | 1.38 | 0.32 | 0.40 | 1.47 | 0.12 | 0.19 | I, R/A |
| K-18 | f | 28 | 3 | 1 | 10 | 2 | 3.38 | 0.39 | 0.28 | 3.50 | 0.27 | 0.30 | I, R/A |
| K-19 | m | 22 | n.d. | n.d. | 11 | 9 | 1.04 | 0.39 | 0.09 | 0.88 | 0.14 | 0.62 | I, R/A |
| K-20 | m | 26 | 3 | 2 | 24 | 11 | 2.17 | 0.15 | 0.04 | 3.26 | 0.39 | 0.46 | I, R/A |
| K-21 | f | 54 | 2 | 0 | 13 | 2 | 0.32 | 0.02 | 0.04 | 0.35 | 0.17 | 0.17 | I, R/A |
| K-22 | f | 29 | 3 | 0 | 31 | 3 | 2.48 | 0.01 | 0.03 | 3.22 | 0.03 | 0.28 | I, R/A |
| K-23 | m | 24 | 3 | 1 | 8 | 0 | 3.60 | 0.08 | 0.12 | 3.51 | 0.03 | 0.08 | I, R/A |
| K-24 | f | 60 | 2 | 0 | 13 | 1 | 0.95 | 0.14 | 0.31 | 1.09 | 0.10 | 0.14 | I, R/A |
|  |  |  |  |  |  |  | **96%** | **33%** | **25%** | **92%** | **25%** | **29%** |  |

Positive reactivity in ELISA is indicated in grey and percentage of positive reactivity is given for each extract or allergen. M, mugwort; R, ragweed; F, feverfew; SPT, skin prick test; I, inhibition ELISA; R/A, ELISA with reduced/alkylated allergens, RBL, mediator release assay, n.d, not determined
