# Supplementary material for: Distinct epitope structures of defensin‐like proteins linked to proline‐rich regions give rise to differences in their allergenic activity
Source: Allergy. 2017 Sep 27;73(2):431–41. doi: 10.1111/all.13298 (PMC5771466; doi:10.1111/all.13298)
Supplement: Supplementary file 3 [file ALL-73-431-s003.docx]

**Distinct epitope structures of defensin-like proteins linked to proline rich regions give rise to differences in their allergenic activity**

Short title: Allergenic defensin-polyproline linked proteins

Isabel Pablos^1^, Stephanie Eichhorn^1^, Yoan Machado^1^, Peter Briza^1^, Alina Neunkirchner^2^, Beatrice Jahn-Schmid^3^, Sabrina Wildner^1,4^, Wai Tuck Soh^1^, Christof Ebner^5^, Jung-Won Park^6^, Winfried F. Pickl^2^, Naveen Arora^7^, Stefan Vieths^8^, Fatima Ferreira^1^, Gabriele Gadermaier^1^

^1^University of Salzburg, Department of Molecular Biology, Division of Allergy and Immunology, Salzburg, Austria

^2^Medical University of Vienna, Institute of Immunology, Center for Pathophysiology, Infectiology and Immunology, Vienna, Austria

^3^Medical University of Vienna, Department of Pathophysiology and Allergy Research, Vienna, Austria

^4^University of Salzburg, Christian Doppler Laboratory for Biosimilar Characterization, Salzburg, Austria

^5^Allergy Clinic Reumannplatz, Vienna, Austria

^6^Yonsei University College of Medicine, Department of Internal Medicine and Institute of Allergy, Seoul, Korea

^7^CSIR-Institute of Genomic and Integrative Biology, Allergy and Immunology Section, Delhi, India

^8^Paul-Ehrlich-Institut, Division of Allergology, Langen, Germany

**Corresponding author**

Gabriele Gadermaier

Department of Molecular Biology, University of Salzburg

Hellbrunnerstraße 34, A-5020 Salzburg, Austria

Telephone: 0043-662-8044-5974, Fax: 0043-662-8044-183

[Gabriele.Gadermaier@sbg.ac.at](mailto:Gabriele.Gadermaier@sbg.ac.at)

**ONLINE MATERIAL AND METHODS**

**In silico structural analyses of Art v 1, Amb a 4 and Par h 1**

Amino acid protein sequences of the defensin domain of Art v 1 (Q84ZX5), Amb a 4 (D4IHC0) and Par h 1 (GenBank KM267639) were aligned using the multiple sequence alignment bioinformatics tool Clustal Omega (1, 2). Three-dimensional structures models of Amb a 4 and Par h 1 were built using SWISS-MODEL software (<https://swissmodel.expasy.org/>) (3-6). The global model quality estimate (GMQE) was considered for the quality of the models. The three-dimensional structure representations were generated with Chimera 1.8 ([www.cgl.ucsf.edu/chimera](file:///C:\Users\Gadermaier\Desktop\Defensin%20paper\2017_02_16\www.cgl.ucsf.edu\chimera)).

**Expression and purification of Art v 1, Amb a 4 and Par h 1**

The mature protein’s genes were cloned into pHisParallel2 vector, Art v 1 and Par h 1 as described (7) and (8), respectively. Amb a 4 was cloned into the same vector and ordered from ATG:biosynthetics GmbH (Merzhausen, Germany). The N-terminus of Amb a 4 sequence was modified by addition of glycine and alanine amino acid for protein expression stability. The expression and purification of the three proteins was performed as previously described (8). Briefly, Art v 1, Amb a 4 and Par h 1 were expressed as non-tagged protein in *E. coli* Rosetta-gamiB (DE3) pLysS (Novagen, Gibbstown, NJ, USA) at 20 °C. Art v 1 was purified by ultrafiltration followed by cation exchange and size exclusion chromatography methods. Amb a 4 and Par h 1 were purified using ammonium sulfate precipitation followed by two steps of hydrophobic interaction and size exclusion chromatography methods. Proteins were stored in 5 mM of ammonium carbonate pH 7.8 at -20°C until further use.

**Physicochemical characterization of purified Art v 1, Amb a 4 and Par h 1**

Purified Art v 1, Amb a 4 and Par h 1 were analyzed by reducing SDS-PAGE using 15% gels. Proteins were visualized with Coomassie Brilliant Blue R-250 staining. Amino acid analysis was performed with all proteins in duplicates following the Pico-Tag method (Waters, Milford, MA, USA). Briefly, phenylthiocarbamyl amino acid derivatives were analyzed by reversed phase high-performance liquid chromatography (UltiMate 3000, Thermo Fischer, Waltham, MA, USA), using a 3.0x150 mm XSELECT™ HSS T3 3.5 µm column (Waters). Hydrolyzed amino acid peaks were quantified at 254 nm by peak area comparison to amino acid standard H (Pierce, Rockford, IL, USA). For intact mass spectrometry measurements, the samples were desalted with C_18_ ZipTips and directly infused into the Q-Exactive mass spectrometer at a flow rate of 1 µL/min, using the nano-electrospray head. Raw data obtained from intact proteins were processed with Protein Deconvolution 2.0. The aggregation behavior of the proteins was analyzed by dynamic light scattering using (DLS) and high performance liquid chromatography (HPLC). The DLS analysis was performed with protein concentration of 1 mg/mL and the data acquisition (10 measurements of 5 seconds duration) was done using DLS802 system (Viscotek Corp). The data processing and calculation of the hydrodynamic radius were performed with the OmniSIZE 3.0 software. The monomeric state of Art v 1, Amb a 4 and Par h 1 was measured by using high performance size exclusion chromatography (HP-SEC). Chromatography was performed on an Ultimate 3000 analytical chromatography system (Dionex Softron GmbH, Germering, Germany) using a Diol-120 column (YMC Europe, Dinslaken, Germany). Samples with a concentration of 3 mg/mL were analyzed at 1 mL/min in a buffer system of 100 mM NaP pH 6.6, 150 mM NaCl, 0.05% NaN3. The molecular weight of Art v 1, Amb a 4 and Par h 1 was estimated by comparing their retention time with those of the standard proteins (Gel Filtration Molecular Weight Markers, Kit for Molecular Weights 6.500 – 66.000 Da, Sigma-Aldrich, St. Louis, MO, USA). The standard proteins were used according to the protocol provided by the company.

**Secondary structure analyses of Art v 1, Amb a 4 and Par h 1**

Secondary structure elements of the recombinant proteins were determined by Fourier-transformed Infrared (FTIR) Spectroscopy using a Tensor 27 (Bruker, Germany) spectrophotometer equipped with an Attenuated Total reflection (Bio-ATR) cell. Data acquisition and analysis were performed using the Opus software v6.5. The measurements were performed with 25 µL of 3 mg/mL protein solution deposited on top of a Bio-ATR cell controlled at 25°C. Absorbance spectra were the average of 120 scans collected over 4000-1000 cm^-1^. The estimation of the secondary structure elements was performed by deconvolution of the amide I band (1700-1600 cm^-1^). A Fourier-self deconvolution algorithm (FSD) was applied to the spectrum over the Amide I band using a Lorentzian shape, bandwidth of 20, deconvolution factor of 2 and a noise reduction of 0.5. FSD spectra were curve-fitted using the Levenberg-Marquardt algorithm. The band positions corresponding to typical secondary structure elements (9) were initially fixed and allowed to vary in position, height and width using the iterative autofit function of the Opus software. The iterations were stopped when the residual Root Mean Square Error was constant and lower than 10^-4^. The estimation of the relative contribution of individual secondary structure elements was obtained from the area of the single bands.

Circular dichroism (CD) spectra were recorded in 5mM of ammonium carbonate pH 7.8 at 25°C with a JASCO J-815 spectropolarimeter (Jasco, Tokyo, Japan). For thermal stability studies the samples were heated from 25°C to 81°C and the spectra were recorded every two degrees. To show the structural changes upon heating, the curves measured at 25°C were subtracted from the rest of the spectra.

**Reduction and alkylation of Art v 1, Amb a 4 and Par h 1**

In order to reduce and alkylate the disulfides bonds of the defensin-like domain, 15 µg vacuum dried proteins were dissolved in 15 µl of extraction buffer E1 from ProteoExtract All-in-One Trypsin Digestion Kit. Subsequently 4 µl of reduction buffer were added to each protein and incubated for 2.5 h at 37°C. For the alkylation, 4 µl of the blocking buffer were added to each protein for 10 min at room temperature. The efficiency of the reduction/alkylation process (i.e. carbamylation of eight cysteines) was monitored by mass spectrometry as described above. Additionally, the folding of the reduced and alkylated proteins was evaluated by CD and FTIR as described above.

**IgE reactivity of pollen extracts and purified recombinant allergens**

Pollen grains from, mugwort *(Artemisia vulgaris)* and ragweed *(Ambrosia artemisiifolia)* were purchased from Allergon AB (Ängelholm, Sweden) and the pollen extracts were prepared as previously described (10). Pollen grains from feverfew *(Parthenium hysterophorus)* was collected and pollen extract was prepared as described (8). To evaluate the IgE reactivity to extracts, 0.2 µg of mugwort, ragweed and feverfew extracts were immobilized onto Maxisorp ELISA plates. Unspecific binding was blocked with 137 mM NaCl, 10 mM Tris pH 7.5, 0.05% (v/v) Tween 20 (TBST) and 1% (w/v) BSA. Sera from weed pollen allergic patients from three different countries (Austria n=36, Canada n=38 and Korea n=24) or healthy donors were diluted 1:4 and incubated overnight at 4°C. For IgE detection, an alkaline phosphatase-conjugated anti-human IgE (BD Biosciences, Franklin Lakes, NJ, USA) was used as secondary antibody. The signal was measured using the substrate 4-nitrophenyl phosphate (Sigma-Aldrich, St Louis, MO, USA) and the measurement was performed at an OD of 405/492 nm. To evaluate the IgE reactivity to the recombinant allergens the same ELISA protocol was followed. In this case, 0.2 µg of purified Art v 1, Amb a 4 and Par h 1 were immobilized onto the plates. Additionally, IgE reactivity of purified allergens with reduced and alkylated disulfide bonds was studied. For this, a chemiluminescence ELISA method was selected. Previously reduced and alkylated Art v 1, Amb a 4 and Par h 1 (0.2 µg) were immobilized onto Lumitrac high binding ELISA plates (Greiner Bio-One, Germany). The same ELISA protocol as above was followed, but a horseradish peroxidase-conjugated monoclonal anti-human IgE (Southern Biotech, Birmingham, AL, USA) as second antibody and BM chemiluminescence ELISA substrate POD (Roche, Germany) were used. Results were obtained by measuring relative chemiluminescent unit (RLU). In all ELISA experiments, four non-atopic human sera (NHS) were used as control and the threshold for positive response was considered as 3*NHS signal.

**IgE cross-reactivity between Art v 1, Amb a 4 and Par h 1**

To study the IgE cross-reactivity between the purified allergens, a cross-inhibition ELISA was performed. Purified Art v 1, Amb a 4 and Par h 1 (0.2 µg) were immobilized onto the plates. Here the sera were pre-incubated overnight at 4°C with 10 µg/ml of the inhibitor, Art v 1, Amb a 4, Par h 1 or blocking buffer (maximum IgE reactivity) and then added to the plates. Patients’ sera used for inhibition assays are indicated in Online Table 1. Results are shown as percentage of inhibition of IgE reactivity.

**Mediator release assay**

The allergenicity of the purified proteins was evaluated using rat basophil degranulation leukemia (RBL) assay as described (11, 12). The RBL-2H3 cell, transfected with the anti-human IgE high affinity receptor, were sensitized with patients’ sera overnight (n=7). The cells were then stimulated with increasing concentration of purified Art v 1, Amb a 4 and Par h 1 and the release of β-hexosaminidase was measured by the enzymatic cleavage of the fluorogenic substrate 4-Methyl umbelliferyl N-acetyl-β-D-glucosaminide (Sigma, Germany). The maximum release was obtained when the cells were treated with 10% of Triton X-100.

**Proteolytic degradation analyzed by endolysosomal proteases and cathepsin S**

The proteolytic stability of the purified allergens to endolysosomal proteases was studied using an endolysosomal degradation assays as previously described (13). Briefly, 5 μg of Art v 1, Amb a 4 and Par h 1 were incubated at different time points with 8 μg of isolated microsomal fraction from the JAWS II cell line in 100 mM citrate buffer pH 4.8 and 2 mM dithiothreitol. The kinetic of the degradation was analyzed by SDS-PAGE, in each time point 2.5 µg of proteins were loaded onto the gels. Additionally, the high molecular weight degradation products and the pool of generated peptides were identified by mass spectrometry as described below.

In addition, recombinant human procathepsin S was expressed, purified, and autoactivated as described (14), with an additional size exclusion chromatography in storage buffer (100 mM NaCl, 5 mM EDTA, 10 mM sodium acetate pH 5.0) after autoactivation. Recombinant Art v 1, Amb a 4 and Par h 1 were dissolved in digestion buffer 100 mM NaCl, 5 mM EDTA, 2 mM DTT and 10 mM sodium acetate pH 4.8. Subsequently, the proteins were incubated with active cathepsins S (molar ratio of 1:20 enzyme:substrate) at 37 °C. The kinetic of the degradation and the degradation products were analyzed by SDS-PAGE and mass spectrometry as described.

**Mass spectrometry of proteolytic degradation**

The pool of peptides generated in the endolysosomal degradation assays was assessed by mass spectrometry using a Q-Exactive mass spectrometer (Thermo Fisher Scientific) with nano-electrospray and nano-HPLC (Dionex Ultimate 3000, Thermo Fisher Scientific). Samples were desalted with C_18_ ZipTips (Millipore) according to the manufacturer’s instructions. After loading the peptides, the column (PepSwift Monolithic Nano Column, 100 µm x 25 cm, Dionex) was developed with an acetonitrile gradient (Solvent A: 0.1% (v/v) formic acid (FA), 0.01% (v/v) trifluoroacetic acid (TFA), 5% (v/v) acetonitrile (ACN); solvent B: 0.1% (v/v) FA, 0.01% (v/v) TFA, 90% (v/v) ACN; 5–45% B in 60 min) at flow rate of 0.8 µL/min at 55°C. For peptide identification, a top 12 method was used, with the normalized fragmentation energy at 27%. Survey and fragment spectra were analyzed with Proteome Discoverer version 1.4 (Thermo Fisher Scientific) and PEAKS Studio 7 (Bioinformatics Solutions, Waterloo, Canada). To minimize false positive results, only peptide identifications with a high or medium confidence score were used for the analyses. Protein degradation maps were generated using the online tools Draw Map 14 (http://ms.biomed.cas.cz/MSTools/DrawMap/DrawMap.php) (15).

**T cell cross-reactivity between Art v 1, Amb a 4 and Par h 1**

Monocyte-derived dendritic cells (mdDCs) were differentiated as described (16, 17) and incubated at 2x105/well in 96-well flat-bottom culture plates with whole recombinant allergens or immunodominant Art v 1_25-36_ peptide (range: 30-0.01 µmol) in a total volume of 100 µl 1x IMDM plus 10% FCS at 37°C overnight. Subsequently, Art v 125-36-specific TCR tg Jurkat T cells (1 x 105/well) (18, 19) were added and co-cultured (total volume 200 µl/well) for 6 h. Subsequently, IL-2 promoter driven luciferase activity was determined (18).

T cell lines and clones specific for Art v 1_25-36_ were established from PBMC derived from mugwort allergic donors as described previously (20). 10^4^ T cells were stimulated in duplicates with Art v 1_25-36_ peptide (3µM), Art v 1, Amb a 4 and Par h 1 at the indicated concentrations in the presence of 10^5^ irradiated autologous PBMC. After 48 hours proliferation was assessed by adding 0.5µCi of ^3^H-thymidine for another 16 hours. Then cells were harvested and ^3^H-uptake measured by scintillation β-counting.

**Data processing and statistical analyses**

Patients’ data (n=30) were clustered according to the IgE reactivity data against the three proteins, the effect of reduction and alkylation on IgE reactivity, and the ability to cross-react with the prototypical defensin-polyproline linked protein Art v 1. An unsupervised cluster analysis was performed with ClustVis, a web tool for visualizing clustering of multivariable data using Principal Component Analysis and heat map. For this, the Ward method with correlation clustering distances for rows was used (21). All statistical analyses were performed in GraphPad Prism. Since three groups were always compared in the ELISA experiments, a Friedman test for non-parametric analyses followed by a Dunn’s Multiple Comparison post-hoc test was used. Statistical significances were reported as follows: *** p ≤ 0.001, ** p ≤ 0.01, * p ≤ 0.05 and ns, not significant. Additionally, a Spearman correlation analysis was performed with the IgE reactivity data where the correlation coefficient (r) is shown.

**REFERENCES**

1. Sievers F, Wilm A, Dineen D, Gibson TJ, Karplus K, Li W, et al. Fast, scalable generation of high-quality protein multiple sequence alignments using Clustal Omega. *Mol Syst Biol* 2011;**7**:539.

2. Goujon M, McWilliam H, Li W, Valentin F, Squizzato S, Paern J, et al. A new bioinformatics analysis tools framework at EMBL-EBI. *Nucleic Acids Res* 2010;**38**(Web Server issue):W695-699.

3. Biasini M, Bienert S, Waterhouse A, Arnold K, Studer G, Schmidt T, et al. SWISS-MODEL: modelling protein tertiary and quaternary structure using evolutionary information. *Nucleic Acids Research* 2014;**42**(W1):W252-W258.

4. Bordoli L, Kiefer F, Arnold K, Benkert P, Battey J, Schwede T. Protein structure homology modeling using SWISS-MODEL workspace. *Nat. Protocols* 2008;**4**(1):1-13.

5. Kiefer F, Arnold K, Kunzli M, Bordoli L, Schwede T. The SWISS-MODEL Repository and associated resources. *Nucleic Acids Res* 2009;**37**(Database issue):D387-392.

6. Guex N, Peitsch MC, Schwede T. Automated comparative protein structure modeling with SWISS-MODEL and Swiss-PdbViewer: a historical perspective. *Electrophoresis* 2009;**30 Suppl 1**:S162-173.

7. Himly M, Jahn-Schmid B, Dedic A, Kelemen P, Wopfner N, Altmann F, et al. Art v 1, the major allergen of mugwort pollen, is a modular glycoprotein with a defensin-like and a hydroxyproline-rich domain. *FASEB J* 2003;**17**(1):106-108.

8. Pablos I, Eichhorn S, Briza P, Asam C, Gartner U, Wolf M, et al. Proteomic profiling of the weed feverfew, a neglected pollen allergen source. *Sci Rep* 2017;**7**(6049).

9. Barth A, Zscherp C. What vibrations tell us about proteins. *Q Rev Biophys* 2002;**35**(4):369-430.

10. Gadermaier G, Eichhorn S, Vejvar E, Weilnbock L, Lang R, Briza P, et al. Plantago lanceolata: an important trigger of summer pollinosis with limited IgE cross-reactivity. *J Allergy Clin Immunol* 2014;**134**(2):472-475.

11. Foetisch K, Westphal S, Lauer I, Retzek M, Altmann F, Kolarich D, et al. Biological activity of IgE specific for cross-reactive carbohydrate determinants. *Journal of Allergy and Clinical Immunology* 2003;**111**(4):889-896.

12. Gadermaier G, Jahn-Schmid B, Vogel L, Egger M, Himly M, Briza P, et al. Targeting the cysteine-stabilized fold of Art v 1 for immunotherapy of Artemisia pollen allergy. *Mol Immunol* 2010;**47**(6):1292-1298.

13. Egger M, Jurets A, Wallner M, Briza P, Ruzek S, Hainzl S, et al. Assessing protein immunogenicity with a dendritic cell line-derived endolysosomal degradome. *PLoS One* 2011;**6**(2):e17278.

14. Freier R, Dall E, Brandstetter H. Protease recognition sites in Bet v 1a are cryptic, explaining its slow processing relevant to its allergenicity. *Sci Rep* 2015;**5**:12707.

15. Kavan D, Man P. MSTools—Web based application for visualization and presentation of HXMS data. *International Journal of Mass Spectrometry* 2011;**302**(1–3):53-58.

16. Pickl WF, Majdic O, Kohl P, Stockl J, Riedl E, Scheinecker C, et al. Molecular and functional characteristics of dendritic cells generated from highly purified CD14+ peripheral blood monocytes. *J Immunol* 1996;**157**(9):3850-3859.

17. Neunkirchner A, Leb-Reichl VM, Schmetterer KG, Mutschlechner S, Kueng HJ, Haiderer D, et al. Human TCR transgenic Bet v 1-specific Th1 cells suppress the effector function of Bet v 1-specific Th2 cells. *J Immunol* 2011;**187**(8):4077-4087.

18. Leb VM, Jahn-Schmid B, Schmetterer KG, Kueng HJ, Haiderer D, Neunkirchner A, et al. Molecular and functional analysis of the antigen receptor of Art v 1-specific helper T lymphocytes. *J Allergy Clin Immunol* 2008;**121**(1):64-71.

19. Jahn-Schmid B, Fischer GF, Bohle B, Fae I, Gadermaier G, Dedic A, et al. Antigen presentation of the immunodominant T-cell epitope of the major mugwort pollen allergen, Art v 1, is associated with the expression of HLA-DRB1 *01. *J Allergy Clin Immunol* 2005;**115**(2):399-404.

20. Jahn-Schmid B, Kelemen P, Himly M, Bohle B, Fischer G, Ferreira F, et al. The T cell response to Art v 1, the major mugwort pollen allergen, is dominated by one epitope. *J Immunol* 2002;**169**(10):6005-6011.

21. Metsalu T, Vilo J. ClustVis: a web tool for visualizing clustering of multivariate data using Principal Component Analysis and heatmap. *Nucleic Acids Res* 2015;**43**(W1):W566-570.
